# Identification and Localization of the Cyclic Nucleotide Phosphodiesterase 10A in Bovine Testis and Mature Spermatozoa

**DOI:** 10.1371/journal.pone.0161035

**Published:** 2016-08-22

**Authors:** Serge Goupil, Loïze Maréchal, Hassan El Hajj, Marie-Ève Tremblay, François J. Richard, Pierre Leclerc

**Affiliations:** 1 Département d’obstétrique, gynécologie et reproduction, Université Laval, et Centre de recherche du CHU de Québec-Université Laval, Québec, Canada; 2 Département de médecine moléculaire, Université Laval, et Centre de recherche du CHU de Québec-Université Laval, Québec, Canada; 3 Département des sciences animales, Université Laval, et Centre de recherche du CHU de Québec-Université Laval, Québec, Canada; 4 Centre de recherche en reproduction, développement et santé intergénérationnelle (CRDSI), Université Laval, Québec, Canada; 5 Centre de recherche du CHU de Québec-Université Laval, G1V 4G2, Québec, QC, Canada; Carl von Ossietzky Universitat Oldenburg, GERMANY

## Abstract

In mammals, adenosine 3’, 5’-cyclic monophosphate (cAMP) is known to play highly important roles in sperm motility and acrosomal exocytosis. It is known to act through protein phosphorylation via PRKA and through the activation of guanine nucleotide exchange factors like EPAC. Sperm intracellular cAMP levels depend on the activity of adenylyl cyclases, mostly SACY, though transmembrane-containing adenylyl cyclases are also present, and on the activity of cyclic nucleotide phosphodiesterases (PDE) whose role is to degrade cAMP into 5’-AMP. The PDE superfamily is subdivided into 11 families (PDE1 to 11), which act on either cAMP or cGMP, or on both cAMP and cGMP although with different enzymatic properties. PDE10, which is more effective on cAMP than cGMP, has been known for almost 15 years and is mostly studied in the brain where it is associated with neurological disorders. Although a high level of *PDE10A* gene expression is observed in the testis, information on the identity of the isoforms or on the cell type that express the PDE10 protein is lacking. The objective of this study was to identify the PDE10A isoforms expressed in the testis and germ cells, and to determine the presence and localization of PDE10A in mature spermatozoa. As a sub-objective, since *PDE10A* transcript variants were reported strictly through analyses of bovine genomic sequence, we also wanted to determine the nucleotide and amino acid sequences by experimental evidence. Using RT-PCR, 5’- and 3’-RACE approaches we clearly show that *PDE10A* transcript variants X3 and X5 are expressed in bovine testis as well as in primary spermatocytes and spermatids. We also reveal using a combination of immunological techniques and proteomics analytical tools that the PDE10A isoform X4 is present in the area of the developing acrosome of spermatids and of the acrosome of mature spermatozoa.

## Introduction

Cyclic AMP (adenosine 3’, 5’-cyclic monophosphate) is an important player in sperm function. It is involved in flagellar movements and motility, and in cellular events that occur in the sperm head, that will lead to successful interaction with the egg and to fertilization [1, 2]. It plays a key role during sperm capacitation [3–6] and the acrosomal exocytosis [7–10], where it affects different intracellular signalling pathways. Through activation of the cAMP-dependent protein kinase (PRKA), cAMP induces an increase in protein phosphotyrosine content [4, 5] through activation of SRC-related tyrosine kinases [11–13] and the ERK (extracellular regulated kinase) signalling pathway [14]. In addition to its PRKA-dependent effects, different studies have clearly demonstrated that cAMP also activates EPAC (Exchange Proteins directly Activated by cAMP) during capacitation and acrosome exocytosis [15–17].

As in somatic cells, sperm intracellular cAMP concentration is regulated by the opposite action of two enzymes: the adenylyl cyclases (AC), which synthesize cAMP from ATP, and the phosphodiesterases (PDE), which break the phosphodiester bond of cAMP to form 5’-adenosine monophosphate (AMP). It has been clearly demonstrated that ADCY10 (soluble adenylyl cyclase; SACY) is the major adenylyl cyclase enzyme present in testicular germ cells and in spermatozoa [18, 19]. Transmembrane-containing AC that can be activated by forskolin are also present in sperm [1, 20, 21]. These AC would be activated by zonae pellucidae, an effect that is mediated by heterotrimeric GTP binding regulatory proteins (G proteins) [20, 21], which supports the defective zona pellucida-induced acrosome reaction observed in mice deprived of ADCY3 [22].

The phosphodiesterase (PDE) superfamily is composed of 11 families of enzymes encoded by more than 20 genes. Considering all the splice variants for some of the gene products, there could be more than 100 PDEs that degrade either cAMP (PDE4, 7 and 8), cGMP (PDE5, 6 and 9), or both (PDE1, 2, 3, 10 and 11) into their 5’-AMP or 5’-GMP forms [23]. PDE family members share homology in their catalytic, C-terminal, domain, while the regulatory domain brings the specificity to PDE families. The latter includes binding sites for cyclic nucleotides, protein-protein interaction domains, and phosphorylation sites, and might be involved in subcellular localization. Over the years the role of PDEs in cellular physiology has been assessed using general non-selective inhibitors such as caffeine, theophylline, pentoxifylline and IBMX (3-isobutyl-1-methylxanthine). Caffeine stimulates sperm respiration, maintains motility, and accelerates sperm capacitation and penetration into eggs [24, 25], and theophylline initiates motility in immature epididymal sperm [26]. Recently, after thawing testicular biopsy, theophylline treatment improved testicular sperm motility, facilitated sperm selection for intra-cytoplasmic sperm injection (ICSI), and increased fertilization, blastulation and pregnancy rates [27]. Although IBMX is a general specific but non-selective PDE inhibitor, it has no effect on PDE8 and PDE9 families.

Initial characterizations revealed that Ca^2+^-calmodulin (CaM)-dependent PDE (PDE1) accounts for the major part of sperm PDE activity [28]. This enzyme is present in the head and tail of rat sperm, mostly associated with the detergent-resistant fraction [29]. In human sperm, PDE1 is found at the equatorial segment of the head as well as the mid and principal piece of the flagellum, while PDE3 is present in the post acrosomal segment of the head [30]. Incubation of bovine or human sperm with specific inhibitors suggests that PDE1 is involved in capacitation and/or acrosomal exocytosis while PDE4 would affect motility [31, 32]. The involvement of PDE4 with motility is in agreement with recent findings suggesting that PDE4 interacts with AKAP3, a scaffold protein found in the flagellum’s fibrous sheath [33].

Transcripts encoding members from most, if not all, PDE families have been detected by PCR amplification of mouse testis and human sperm RNA [34, 35] and members of some PDE families were detected immunologically in sperm [35–37]. PDE10, one of the latest identified PDE, was detected among the different families found in sperm. It has been cloned and sequenced in the late 90’s and its transcript is present at high levels in the brain and testis [38–40]. PDE10 is involved in diseases such as the neurodegenerative Huntington’s disease [41], psychosis [42] and schizophrenia [43]. Although it acts on both cAMP and cGMP, its affinity for cAMP is more than 25 times higher than for cGMP [38, 40]. PDE10 is encoded by a single gene (*PDE10A*), but, as a result of alternative splicing, different isoforms with specific N- and C-termini can be found [44].

Although PDE10A has been previously detected in the testis and in epididymal spermatozoa of mice and dogs [35, 36], the identity of the specific isoform present in this tissue and cells remains to be clearly determined. Since it is mostly the N-terminal domain of the protein that differs among PDE10A isoforms and since it is within these domains that post-translational modifications affecting the subcellular localization occur, our objective was to identify the PDE10A isoforms present in mature sperm in order to further investigate the importance of these enzymes in sperm function.

For bovine PDE10A, only predicted nucleotide and deduced amino acid sequences obtained by automated computational analyses of the genomic sequence of the 9^th^ chromosome are available. As of December 31^st^ 2014, there were 5 predicted bovine *PDE10A* nucleotide sequences: transcript variant X1 (accession number XM_010799051; isoform X1), transcript variant X2 (accession number XM_010808740; isoform X2), transcript variant X3 (accession number XM_005211114; isoform X2), transcript variant X4 (accession number XM_010808741; isoform X3), transcript variant X5 (accession number XM_005211116; isoform X4), and a 6^th^ sequence (May 2010) was also found, accession number DAA25953, corresponding to none of the preceding splice variant. From these 6 nucleotide sequences, the amino acid sequences of 5 isoforms can be deduced, with 4 different N-termini. When compared to the isoform X2, the isoform X3 lacks 16 amino acids (AA 281–297). On January 26^th^ 2016, the nucleotide sequences of the 5 predicted transcript variants were removed from public databank and were replaced by two other predicted sequences (accession numbers XM_015464896 & XM_015472936) that encode for the same protein, which correspond the former isoform X2.

It is therefore important to determine experimentally the exact sequences encoding PDE10A isoforms and, for our study, to identify which isoforms are present in bull testis and sperm. As mature sperm are devoid of transcriptional and translational activities, our first approach was to assess the nucleotide sequence encoding the PDE10A isoforms present in the testis.

## Materials & Methods

### RNA isolation, cDNA synthesis and PCR amplification

Bovine testes were obtained from a local slaughterhouse (Abattoir Bolduc, Buckland, QC, Canada) and brought on ice to the laboratory within 2 hr after animal death. Pieces smaller than 1 cm^3^ were cut out underneath the tunica albuginae and kept frozen at -80°C until use. Total RNA was isolated according to the manufacturer’s instruction (RNEasy Mini Kit, Qiagen Inc, Mississauga, ON, Canada), and was next used for 5’- or 3’-RACE using the RLM-RACE Kit (Ambion, Life Technologies Inc, Burlington, ON, Canada). Two conditions of reverse transcription (RT) were used to ensure successful 5’- or 3’- rapid amplification of cDNA ends (RACE) after treatment of 10 μg of total RNA with Calf Intestinal Alkaline Phosphatase, Tobacco Acid Pyrophosphatase, and ligation to an RNA adapter oligonucleotide (5’-RACE adapter, Table 1). For the first condition, RT was done exactly as per the manufacturer’s protocol using the enzymes and reagents from the kit; for the second condition, RNA was reverse transcribed using Superscript III and polyd(T)_12-18_ (both from InVitrogen, Life Technologies) as the starting oligonucleotide primer. 5’-RACE was performed with 1 μl of first strand cDNA using 200 μM dNTPs, 0.3 μM each of the 5’-RACE outer primer from the kit (Table 1) and the PDE10A-out 1R primer (Table 1) in a final reaction volume of 50 μl containing 2.6 units of DNA polymerase (Expand High Fidelity PCR System, Roche Applied Science, Laval, QC, Canada). Polymerase Chain Reaction (PCR) proceeded for 35 cycles (15 s denaturation at 94°C, 30 s annealing at 60°C and 2 min elongation at 72°C) after an initial 2 min denaturation step at 94°C. This was completed by a 7 min elongation step at 72°C. A nested PCR amplification was done under similar conditions except that the gene specific primer was changed for PDE10A-in 1R (Table 1). For the 3’-RACE, DNAse I-treated RNA was reverse transcribed using the 3’-RACE adapter oligonucleotide from the RLM-RACE kit that included a poly d(T)_12_ stretch, and either the Superscript III reverse transcriptase or the M-MLV reverse transcriptase from the kit with similar results. PCR was next done using the PDE10A-for4 (Table 1) primer and the 3’-RACE outer primer from the kit (Table 1), with the Expand Long Template PCR System (Roche) as DNA polymerase. A nested amplification was done replacing the gene specific primer by the PDE10A-for5 primer (Table 1). The size of the amplicons was determined by electrophoresis on agarose gels; the bands of interest were purified and cloned into pGEM-T easy vector (Promega, Madison, WI) and transfected into DH5α cells for amplification and sequencing. Sequence identities were established by searching the *Bos taurus* database using BLAST (http://www.ncbi.nlm.nih.gov).

**Table 1:** Oligonucleotide primers used for PCR amplification.

PDE10A-out 1R	5’-GCA AAC ACA GCA CAG ACT GG-3’
PDE10A-in 1R	5’-ATT CCA GGC GGG ATA AAC ACA-3’
PDE10A-for4	5’-CTT TGA CGT TGG CCC CTT TG-3’
PDE10A-for5	5’-ATG GAG CAG CAC CAC TTC TC-3’
5’-RACE outer primer	5’-GCT GAT GGC GAT GAA TGA ACA CTG-3’
5’-RACE inner primer	5'-CGC GGA TCC GAA CAC TGC GTT TGC TGG CTT TGA TG-3'
3’-RACE outer primer	5’-GCG AGC ACA GAA TTA ATA CGA CT-3’
3’-RACE inner primer	5'-CGC GGA TCC GAA TTA ATA CGA CTC ACT ATA GG-3'
PDE10AX3-for	5’-ATG AGC CAT GAG CCA GCG GAA-3’
PDE10AX5-for	5’-ATG ACT TTC TGT GGG ATG GCA A-3’
PDE10AX35-rev	5’-TCA GTC ATC GGT CTT CAC G-3’
5’-RACE adapter	5'-GCU GAU GGC GAU GAA UGA ACA CUG CGU UUG CUG GCU UUG AUG AAA-3'
3’-RACE adapter	5'-GCG AGC ACA GAA TTA ATA CGA CTC ACT ATA GGT_12_ VN-3'

PDE10A gene-specific primers were designed within the Open Reading Frame of the predicted coding sequence that is identical for PDE10A and the 5 reported transcript variants (X1 to X5; accession numbers: XM_010799051, XM_010808740, XM_005211114, XM_010808741, XM_005211116), and another reported variant TPA accession number: DAA25953.

Standard PCR amplifications for the open reading frame of specific PDE10A transcript variants 3 and 5 were done using the primer combination PDE10AX3-for with PDE10AX35-rev, and PDE10AX5-for with PDE10AX35-rev (Table 1), respectively. These experiments were done on total RNA isolated from testis as well as from haploid (1n) and tetraploid (4n) germ cells that was reverse transcribed with Superscript III to confirm the presence of these PDE10A variants.

### Isolation of bull testicular germ cells

Haploid (1n, 1c) and tetraploid (2n, 4c) germ cells were isolated from bull testis as previously described [45]. A piece of bull testis (2 cm x 2 cm x 1 cm) was taken underneath the tunica albuginae and rinsed in D-PBS (137 mM NaCl, 2.7 mM KCl, 0.9 mM CaCl_2_, 0.5 mM MgCl_2_, 1.5 mM KH_2_PO_4_, 8.1 mM Na_2_HPO_4,_ pH 7.4) to get rid of the blood. The volume was adjusted to 15 ml with D-PBS and the biopsy was minced with scissors. The cells were then treated with 0.1% trypsin (Invitrogen) for 10 min at 37°C without shaking before addition of 150 mM MgCl_2_ and 30 μg deoxyribonuclease I (DNAse I, Sigma, St-Louis, MO) and further incubation at 37°C for another 10 minutes with shaking. Fetal bovine serum (1.5 ml) was next added and the seminiferous tubules were dissociated by pipetting. The preparation was filtered on a 70 μm nylon mesh strainer (Becton-Dickinson, Bedford, MA) to recover the isolated cells. The debris were rinsed with 15 ml D-PBS, vortexed and filtered again. The cells were centrifuged for 15 minutes at 250 x g, the pellet was resuspended in 15 ml D-PBS supplemented with DNAse I, and centrifuged for another 15 minutes at 250 x g. The resulting pellet was resuspended in Isoton II solution (167 mM NaCl, 5 mM KCl, 1 mM EDTA, 0.1% BSA, 10 mM HEPES pH 7.0). Hoescht 33342 (Sigma) was added to a 5 μg/ml final concentration, and the cells were incubated at 37°C for 1 hour before sorting according to their DNA content. Cell sorting was performed using a fluorescence-activated cell sorter (FACS; Epics Elite ESP, Beckman Coulter, Miami, FL; equipped with a HeCd laser, Omnichrome Model 100, Omnichrome, Chino, CA) with an excitation wavelength of 325 nm.

### Sperm preparation and subcellular fractioning

Freshly ejaculated bull semen was collected at an artificial insemination facility (CIAQ Inc., St-Hyacinthe, QC) and kindly donated by l'Alliance Semex. The semen was transported to the laboratory at 18°C, arriving within 2.5 h after collection. Upon arrival, 1 ml was diluted with 10 ml of D-PBS and spermatozoa were washed from the seminal plasma by centrifugation (500 x g, 10 min). Sperm cells were washed two more times in 10 ml D-PBS by centrifugation under the same conditions. After the third wash, the supernatant was discarded and spermatozoa were resuspended in 5 ml D-PBS containing protease inhibitors (final concentration: 10 μg/ml each of leupeptine, aprotinine, and pepstatin A, 250 μM PMSF, and 1 mM EDTA) and their concentration was evaluated using a hematocytometer. In some experiments, sperm subcellular fractions were prepared. Plasma membranes were detached from sperm cells by nitrogen cavitation (750 psi for 10 min at 4°C). The suspension was centrifuged once (10,000 x g, 15 min, 4°C) to pellet the demembranated sperm, and the supernatant was centrifuged at 18,000 x g (20 min, 4°C) to remove small cellular debris. The resulting supernatant was subjected to ultracentrifugation at 100,000 x g for 1 h (4°C). The pellet obtained contained sperm membranes whereas the supernatant contained the cytosolic fraction. Proteins from this latter fraction were concentrated by centrifugation using a microcon device (10 kDa cut-off; Millipore, Billerica, MA, USA). The demembranated spermatozoa obtained from the 10,000 x g centrifugation, were resuspended in 1.5 ml D-PBS with protease inhibitors and then subjected to sonication (2 times, 30 s each on ice) to dislocate the heads from the flagellae and to detach other sperm membranes. This suspension was centrifuged for 15 min (4°C) at 1000 x g to pellet the dislocated heads and tails, and the supernatant was centrifuged at 18,000 x g (20 min, 4°C) and then the resulting supernatant ultracentrifuged at 100,000 x g (1 h, 4°C) to pellet other residual sperm membranes. The pellet from the 1000 x g centrifugation was resuspended with 3 ml of 1.6 M sucrose in D-PBS containing 250 μM PMSF, layered on the top of a discontinuous sucrose gradient (1 ml each of 1.8 M and 2.2 M made in D-PBS/PMSF) and subjected to ultracentrifugation at 23,000 x g for 35 min at 4°C. The heads were collected from the pellet and flagellae from the two interfaces. They were next diluted in D-PBS/PMSF and washed by centrifugation at 18,000 x g for 30 min. Protein concentration from each subcellular fraction was evaluated using a micro BCA protein assay kit (Pierce, Rockford, Il, USA) after precipitation with trichloroacetic acid (TCA).

### Western blot procedure

Sperm proteins were solubilized in electrophoresis sample buffer (2% SDS, 10% glycerol, 50 mM DTT, 62.5 mM Tris-HCl, pH 6.8) and heated at 100°C for 5 min, resolved by SDS-PAGE [46] and transferred onto PVDF membrane (Immobilin-P; Millipore, Bedford, MN, USA) [47]. Non-specific sites on the membrane were blocked with 5% (w/v) skimmed milk in Tris-buffered saline containing Tween 20 (TBS-T; 150 mM NaCl, 20 mM Tris-HCl, 0.1% Tween 20, pH 7.4). The membrane was next incubated with a monoclonal anti-PDE10A antibody (clone 1C9, recommended by the manufacturer for western blot; Origene Technologies, Rockville, MD) for 16 h at 4°C. Following several washes, the membrane was incubated with a goat anti-mouse IgG secondary antibody conjugated to horseradish peroxidase for 1 h (GE Healthcare Bio-Sciences Inc., Baie d’Urfé, QC, Canada or Jackson Immunoresearch, West Grove, PA). After five washes in TBS-T, positive immunoreactive bands were detected using an enhance chemiluminescence kit (ECL Prime; GE Healthcare Bio-Sciences Inc.) according to the manufacturer’s instructions and film exposure (Fuji, Tokyo, Japan), or with ECL clarity chemiluminescence kits (Bio-Rad, Mississauga, ON, Canada) and then scanned with the ChemiDoc MP Imaging System (Bio-Rad).

### Immunoprecipitation

Washed spermatozoa were lysed in immunoprecipitation (IP) buffer (100 mM NaCl, 50 mM Pipes, pH 6.8, 1 mM EDTA, 1 mM EGTA, 0.2 mM MgCl_2_ containing 0.75% Dodecyl-β-D-Maltopyranoside [DDM; Carbosynth Ltd, Berkshire, UK]) for 30 min at 4°C. The sample was next centrifuged at 18,000 x g (20 min). The supernatant was collected and pre-cleared by the addition of 2.5 μg of commercial non-immune mouse IgG, incubated for 1 h at 4°C with continuous rotation, followed by the addition of Protein G-coated sepharose beads (Amersham, Uppsala, Sweden) and incubated for another 1 h with continuous rotation. The pre-cleared fraction was obtained after removal of the beads by centrifugation (4,500 x g, 3 min). Monoclonal anti-PDE10A antibody (5 μg; clone 1C9, Origene) was added to the pre-cleared fraction and incubated for 2 h. Protein G-coated sepharose beads were added and the suspension was incubated for another 2 h with constant rotation. The beads were pelleted by centrifugation, washed 3 times with 1 ml cold (4°C) IP buffer and 2 more times with cold (4°C) NH_4_CO_3_ (25 mM). The final pellet was sent for identification by LC-MS/MS at our Research Centre’s Proteomic core facility (Proteomics platform of the Eastern Quebec Genomics Center; Quebec, QC, Canada).

### Proteomic analysis

Tryptic digestion was performed on a MassPrep liquid handling robot according to the manufacturer’s specifications and to the protocol of Shevchenko et al. [48] with the modifications suggested by Havlis et al. [49]. Briefly, proteins were reduced with 10 mM DTT and alkylated with 55 mM iodoacetamide. Trypsin digestion was performed using 105 mM of modified porcine trypsin at 58°C for 1 h. Digestion products were extracted using 1% formic acid, 2% acetonitrile followed by 1% formic acid, 50% acetonitrile. The recovered extracts were pooled, vacuum centrifuge dried, then resuspended into 8 μl of 0.1% formic acid and 4 μl were analyzed by mass spectrometry. Peptide samples were separated by online reversed-phase (RP) nanoscale capillary liquid chromatography (nanoLC) and analyzed by electrospray mass spectrometry (ES MS/MS). The experiments were performed with a Thermo Surveyor MS pump connected to a LTQ linear ion trap mass spectrometer equipped with a nanoelectrospray ion source. Peptide separation took place on a PicoFrit column BioBasic C18, 10 cm x 0.075 mm internal diameter, with a linear gradient from 2–50% solvent B (acetonitrile, 0.1% formic acid) in 30 min, at 200 nL/min (obtained by flow-splitting). Mass spectra were acquired using a data dependent acquisition mode using Xcalibur software version 2.0. Each full scan mass spectrum (400 to 2000 m/z) was followed by collision-induced dissociation of the seven most intense ions. The dynamic exclusion (30 sec exclusion duration) function was enabled, and the relative collisional fragmentation energy was set to 35%. All MS/MS samples were analyzed using Mascot set up to search the Eukariata database. Mascot was searched with a fragment ion mass tolerance of 0.50 Da and a parent ion tolerance of 2.0 Da. Iodoacetamide derivative of cysteine was specified as a fixed modification and oxidation of methionine was specified as a variable modification. Two missed cleavage were allowed. MS/MS based peptide and protein identifications were validated using Scaffold software. Peptide identifications were accepted if they could be established at greater than 95.0% probability as specified by the Peptide Prophet algorithm [50]. Protein identifications were accepted if they could be established at greater than 95.0% probability and contained at least 2 identified peptides. Protein probabilities were assigned by the Protein Prophet algorithm [51]. Proteins that contained similar peptides and could not be differentiated based on MS/MS analysis alone were grouped to satisfy the principles of parsimony.

### Immunofluorescence

Sperm washed in D-PBS as described above were resuspended at 20 x 10^6^ spz/ml. Thirty-five microliter of the suspension were put on poly-L-lysine coated coverslips and allowed to settle at room temperature for 30 min. Sperm on the coverslips were fixed for 15 min at room temperature with 3.7% formaldehyde, washed 5 times with PBS, then permeabilized for 10 min with 0.2% Triton X-100. The coverslips were blocked for 1 h with PBS containing 1% BSA, then incubated overnight at 4°C with a monoclonal anti-PDE10A antibody (clone 3G9, recommended by the manufacturer for immunofluorescence; Origen). After extensive washes in PBS, spermatozoa were incubated with a DyLight 594 conjugated goat-anti mouse IgG (Pierce). The coverslips were then submitted to extensive washes in PBS and finally mounted on slides with DABCO (220 mM 1,4diazabicyclo[2,2,2]octane in 90% glycerol) as an antibleaching agent. In some experiments, to assess sperm acrosomal status, the coverslips were incubated for 30 min with 40 μg/ml of FITC-conjugated *Arachis hypogaea* agglutinin (PNA-FITC), and washed extensively in PBS before mounting on slides with DABCO. Spermatozoa were observed by epifluorescence microscopy with proper filters to acquire PDE10A (red) and outer acrosomal membrane (green) signals.

### Immunohistochemistry

Bull testes were rapidly fixed by injecting 25 to 50 ml of bouin’s fixation solution through the tunica albuginae. Small pieces of testes were excised and incubated overnight at 4°C in bouin’s solution. Tissues were next washed with formalin to decrease the yellow coloration, dehydrated through bathing in increasing ethanol concentration and mounted in paraffin blocks. Immunohistochemistry was performed on 6 μm-thick sections. The slides were deparaffinized and rehydrated through successive incubation in ethanol baths with decreasing concentrations. The sections were washed in PBS (137 mM NaCl, 8.1mM Na_2_HPO_4_, 2.7 mM KCl and 1.5 mM KH_2_PO_4_) and endogen peroxidase activity was inhibited with 3% H_2_O_2_ in methanol for 10 min. Antigen retrieval was achieved as routinely done in our laboratory by boiling the slides for 10 min in 10 mM sodium citrate pH 6.0 and non-specific sites were blocked in a 2-step procedure: slides were first incubated for 30 min with 10% goat serum in PBS and next with 1% BSA in PBS for 1 h. The slides were next incubated overnight (4°C) with a monoclonal anti-PDE10A antibody (clone 3G9, Origene) diluted in PBS/BSA. After several washes with PBS containing 0.05% Tween 20, the sections were incubated with a biotinylated goat anti-mouse IgG for 1 h at room temperature, washed with PBS/Tween 20, and processed with Vectastin ABC kit (Pierce). The immune complex was then revealed with 3,3’Diaminobenzidine (DAB; Sigma) and counterstained with Gill’s hematoxylin. Tissue sections were finally mounted in mowiol and observed by light microscopy. Testis sections incubated with the same concentration of commercial non-immune mouse IgG were used as negative controls.

### Immunoelectron microscopy

As indirect immunofluorescence does not discriminate between PDE10A localization at the plasma membrane, at the outer or inner acrosomal membranes, or within the acrosome, we next performed pre-embedding immunocytochemical electron microscopy based on a well-established protocol [52] that we slightly modified to localize precisely PDE10A in the sperm acrosomal region. Freshly ejaculated washed spermatozoa were fixed (125 x 10^6^ spermatozoa per sample) with 1 ml of acrolein 3.5% for 15 min at room temperature, and then washed twice by quick centrifugation with PBS. The pellet was covered with 125 μl of melted 4% agarose (in D-PBS) and gently mixed to let agarose enter the pellet. The sample was solidified at 4°C. Sections of 50 μm were obtained with a vibratome and washed three times in PBS for 10 min each. The sections were incubated in 0.1% sodium borohydride (NaBH_4_) in PBS for 30 min (to reduce remaining aldehyde from the acrolein fixation step) and washed three times in PBS for 10 min each before being blocked for two hours in PBS containing 10% fetal bovine serum, 3% BSA and 0.01% Triton X-100. After 5 washes (5 min each) in Tris buffered saline (TBS; 50 mM Tris pH 7.4, NaCl 150 mM) the sections were incubated overnight at 4°C with a monoclonal anti-PDE10A antibody (clone 3G9, Origene). After five washes (5 min each) in TBS, the sections were incubated overnight at 4°C with the secondary antibody (1.4 nm gold particles-coupled goat anti-mouse IgG, Nanoprobes, Yaphank, NY, USA). The sections were washed three times for 5 min each with TBS, then twice (5 min each) with 3% sodium acetate. Using a silver enhancement kit (HQ silver, Nanoprobes), the staining was revealed at room temperature for 1 min and then rinsed quickly with sodium acetate followed by three 5-min washes with PBS. The sections were post-fixed with osmium tetroxide, dehydrated with sequential alcohol baths and propylene oxide before flat embedding in Durcupan between two ACLAR sheets as described in [53]. The regions of interest were cut and glued on a resin block. Ultrathin sections of ~70 nm were obtained with a Leica UC7 ultramicrotome and collected on mesh grids. Observations were made with FEI Tecnai Spirit G2 transmission electron microscope at 80 kV.

## Results

### Expression of PDE10A in bovine testis

**Fig 1:**
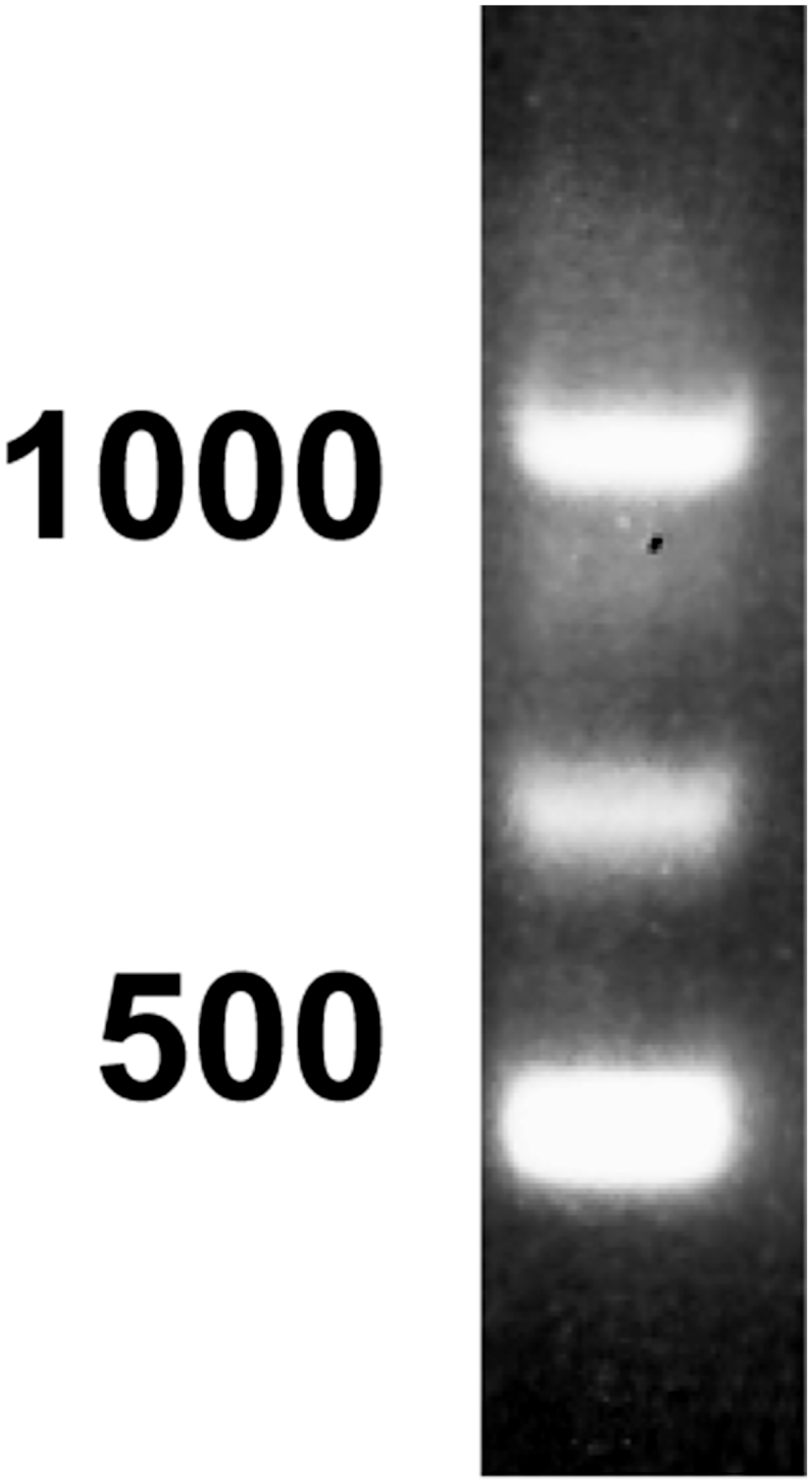
5’-RACE of cDNA encoding PDE10A in bull testis. Total RNA isolated from bull testes was reverse transcribed and processed for 5’-RACE as described in Materials & Methods. The size of the amplified products was visualized after electrophoresis on an agarose gel. Three major amplicons of ≈1100, 700 and 450 bp were obtained. The position of the 1000 and 500 bp standards is indicated on the left. Similar results were obtained with RNA isolated from the testes of 3 different bulls.

Using the 5’-RLM-RACE to determine what *PDE10A* transcript variants and, therefore, protein isoforms are present in bull testis, 3 amplicons of ≈1100, 700 and 450 bp were obtained after electrophoresis on an agarose gel (Fig 1). Each band was next extracted from the gel, cloned, amplified, and its nucleotide sequence determined. For each of the three bull testis RNA samples, the plasmid from several clones (minimum of 6) from each extracted band was analysed. From the sequence obtained, the higher band was 1058 bp-long, including the RNA adapter from the RLM-RACE kit, and encoded the variant X3 of *PDE10A* (nt 1–1003). Our sequence contained 17 additional nucleotides in the 5’ untranslated region when compared to the predicted sequence obtained by computational analysis of the bovine genome (S1 Fig). When our sequence was compared with the bovine genomic sequence (Bos taurus breed Hereford chromosome 9 genomic scaffold, Bos_taurus_UMD_3.1.1, whole genome shotgun sequence, Sequence ID: NW_003104261.1) there were 7 domains that aligned perfectly (Table 2), corresponding putatively to 7 exons.

**Table 2:** Alignment with the genomic sequence.

Genomic	649948–649422	615198–615064	507765–507459	447490–447361	404608–404580	383753–383633	383142–383093	372111–372683
Variant X3	1–527	527–662		663–791	792–820	821–941	942–991	992–1020
Variant X5			1–307	308–437	438–466	467–587	588–637	638–666

Alignment of the nucleotide sequences obtained in our 5’-RLM experiments with the bovine genomic sequence (Bos taurus breed Hereford chromosome 9 genomic scaffold, Bos_taurus_UMD_3.1.1, whole genome shotgun sequence, Sequence ID: NW_003104261.1). The numbers displayed correspond to the nucleotide position within the genomic sequence or within the sequence of the clones obtained in our experiments.

When the mid-size band was cloned, several differences were obtained among the clones. A 704 bp long clone, including the RNA adapter, encoded the *PDE10A* transcript variant X5 (nt 1–539; S2 Fig). When compared with the genomic sequence, our sequence perfectly matched 6 domains, 5 of which being identical to those obtained with the clones corresponding to the transcript variant X3 (Table 2). The first 307 nucleotides in the 5’ end aligned with a genomic domain (putative exon) located between the 2^nd^ and 3^rd^ putative exon of the transcript variant X3. We also obtained 717 nucleotide-long clones (with RNA adapter) that corresponded to transcript variant X5 although they lacked a stretch of 5 nucleotides at nt 176–180 from the variant X5 sequence (GGTAT, S3 Fig) in the open reading frame. This 5 nucleotide gap occurs immediately before the end of the 1^st^ coding exon of the transcript variant X5, resulting, upon translation, in a starting Methionine residue 68 amino acids later in a region of the protein identical among all the PDE10A isoforms. The absence of these 5 nucleotides was also observed in several 674 nt-long clones obtained from the RNA of 2 other bull testes. Again, the sequence was identical to *PDE10A* variant X5 with an initial Met residue occurring 68 amino acids later. From the RNA isolated from one bull testis, we obtained several 552 nt-long clones encoding *PDE10A* transcript variant X5 that lacked a 172 nt portion that also included the GGTAT sequence absent from the previous clones (nt 9–180 from X5; S4 Fig). As for the clones with a 5-nucleotide gap, the initial Met residue occurred 68 amino acids downstream of the published PDE10A X4 isoform initiating codon. Other clones from one bull testis were 735 nt-long and they lacked the GGTAT sequence (nt 176–180 from transcript variant X5) but had a 61-nucleotide insertion between nt 510 and 511 from *PDE10A* variant X5 (S5 Fig). As for the previous clones, the 5-nt gap led to a change in initiation Met residue, but the 61-nt insertion, which is found in the genomic sequence between the last two coding domains identified above for X3 and X5 transcript variants, caused a premature stop codon. Therefore, this clone putatively encodes a 57 amino acid-long protein.

When the smallest band obtained by RLM-RACE was cloned, several clones of 511 and 424 nucleotides long were obtained. The first 113 nucleotides of the 511 nt-long clones did not show homology to any RNA or cDNA sequence in the databases, although the remaining sequence was 358/360 identical to *PDE10A* variant X4, X3, and X2, and 357/359 identical to X5. For the 424 nt-long clones, the first 22 nucleotides showed no homology to any sequence, but the remaining sequence was 363/364 identical to X5, and 359/359 identical to X4, X3, and X2.

**Fig 2:**
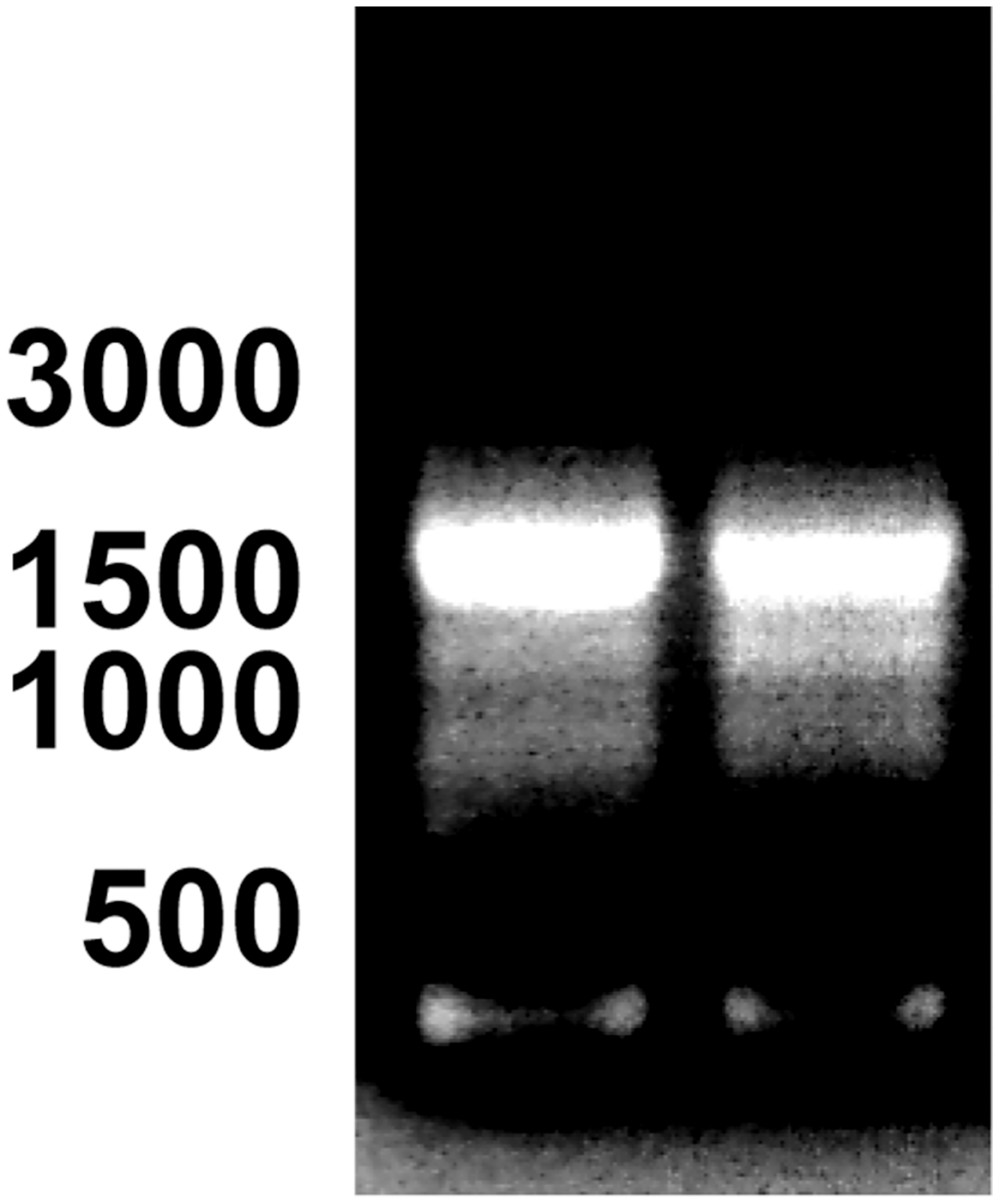
3’-RACE of cDNA encoding PDE10A in bull testis. Total RNA isolated from bull testes was reverse transcribed and processed for 3’-RACE as described in Materials & Methods. The size of the amplified products was visualized after electrophoresis on an agarose gel. The results showing one major amplicon of ≈1500 bp were obtained with 2 different bulls. The position of 500, 1000, 1500 and 3000 bp is indicated on the left. Similar results were obtained using RNA from the testis of another bull.

Since two different PDE10A C-termini have been described in mouse and human, we performed 3’-RACE experiments to determine whether one or more C-terminal ends of PDE10A exists in bull testis. We designed a gene specific oligonucleotide primer that is present in all the predicted *PDE10A* transcript variants in the database (Table 1). After reverse transcription of the testis RNA with the 3’-RACE adapter containing a poly d(T)_12_ stretch (Table 1), we used our specific primer and the 3’-RACE outer and inner primers provided with the kit to obtain a major amplicon of 1512 bp (Fig 2). After extraction of the band from the agarose gel and cloning, the sequence revealed an almost complete identity (1467/1468) with the predicted sequence of *PDE10A* transcript variants X1-X5 (nt 1885–3352 of X5; nt 2349–3816 of X3). In all the clones sequenced from the RNA isolated from different bull testes, the C^3328^ of X3 (C^2864^ of X5) was replaced by a T. When our sequence was compared with the genomic sequence, it aligned perfectly with 6 domains, which putatively correspond to 6 exons.

**Fig 3:**
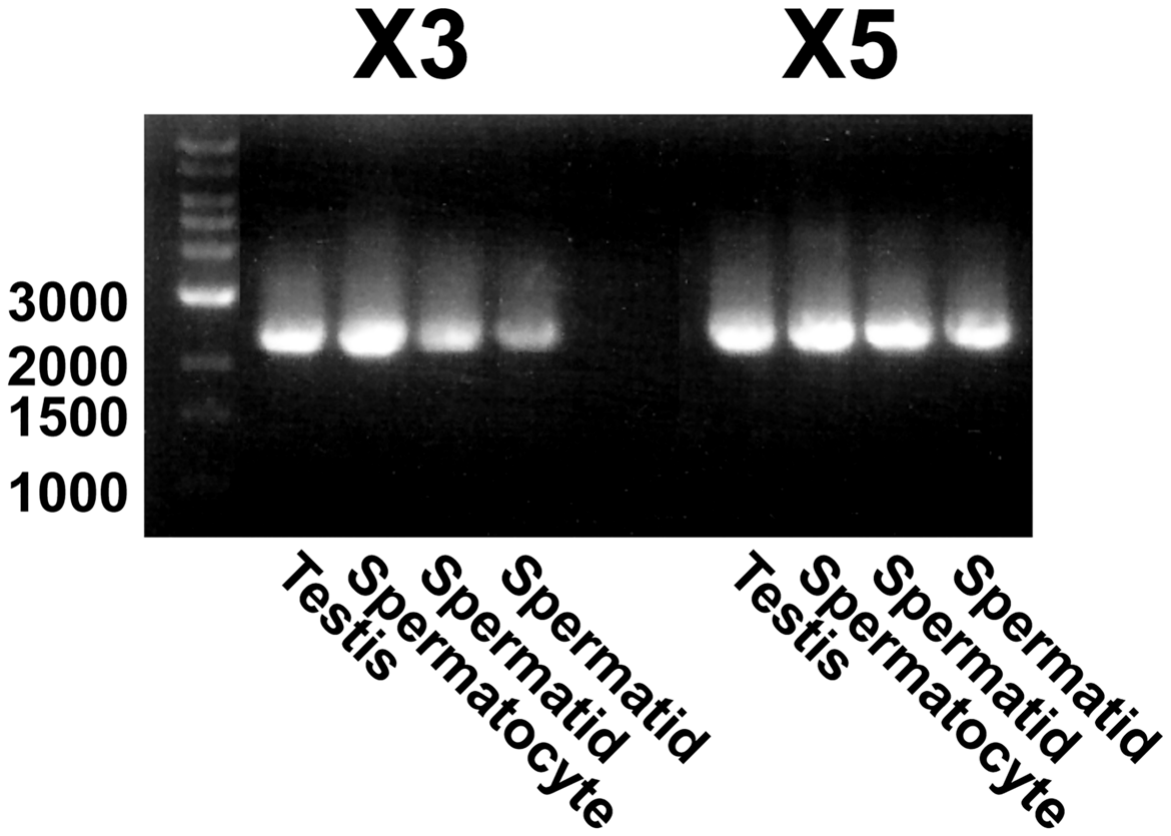
PCR amplification of cDNA encoding PDE10A variants X3 and X5 in bovine testis and spermatogenic germ cells. RNA isolated from bovine testis, testicular tetraploid (spermatocytes) and haploid (spermatids) germ cells was reverse transcribed and the transcripts encoding PDE10A variant X3 and X5 were amplified by PCR using specific primers. Amplification products obtained with spermatid RNA from 2 different bulls are shown. Similar results were obtained with RNA isolated from the testis, spermatocytes and spermatids from another bull. The expected size of the amplicons is 2367 bp and 2349 bp for variants X3 and X5, respectively. The position of molecular weight standards (bp) is shown on the left.

Our results therefore suggest that *PDE10A* transcript variants X3 and X5 are present in the testis. Since the testis contains not only germ cells that will ultimately produce spermatozoa, but also somatic cells such as Sertoli cells, Leydig cells, as well as macrophages and myoid cells, we next attempted to determine whether testicular germ cells express *PDE10A* and whether they express variant X3, X5, or both. Primers were designed to amplify the entire coding sequences of the variants X3 and X5 in RNA isolated from testis, and from primary spermatocytes (2n, 4c) and spermatids (1n, 1c) isolated by flow cytometry according to their DNA content. As shown in Fig 3, both spermatids and spermatocytes express *PDE10A* variant X3 and X5, which would encode the isoforms X2 and X4 of the PDE10A enzyme, respectively.

**Fig 4:**
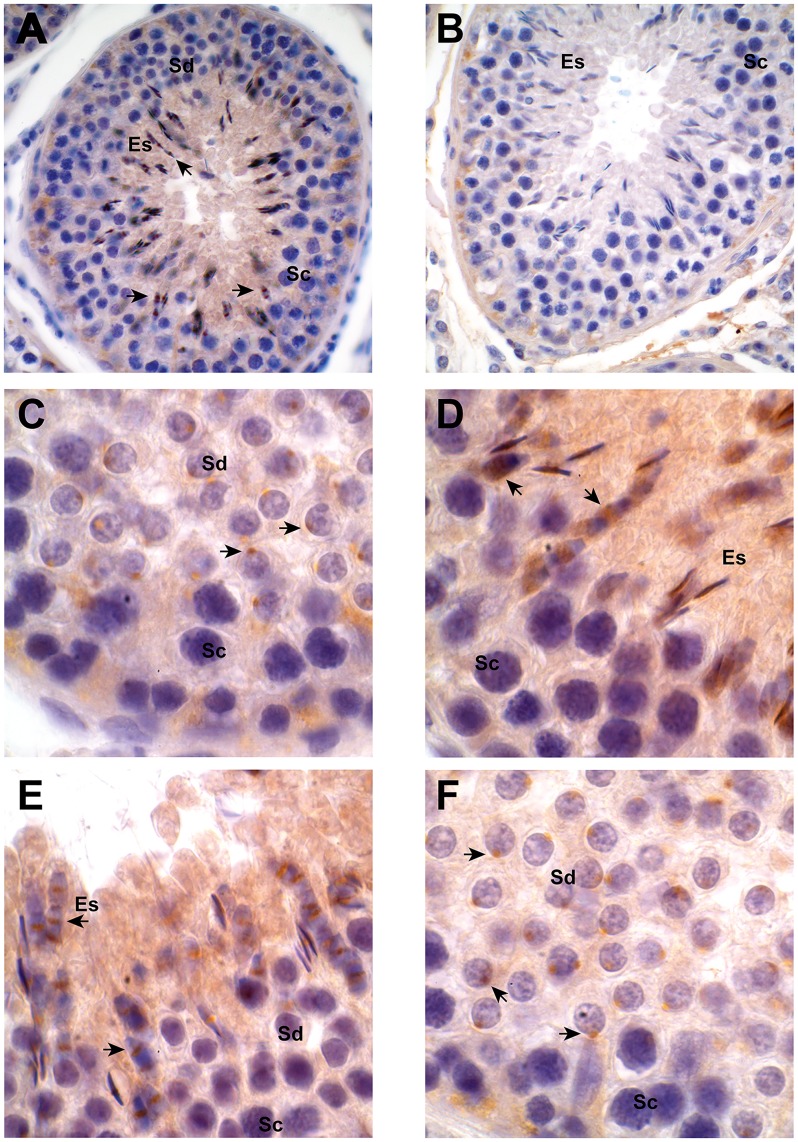
Localization of PDE10A in bovine testis. Bull testicular sections were processed for immunohistochemistry as detailed in Materials & Methods using a monoclonal anti-PDE10A antibody (3G9; Panel A, C, D, E, and F) or non-immune commercial mouse IgG (Panel B). Positive signal (arrows in all Panels) appears as a brownish precipitate. Panels A and B show 400X magnification and Panels C—F show 1000X magnification. Sc stands for primary spermatocytes, Sd stands for round spermatids, and Es stands for elongated spermatids.

The presence of the enzyme PDE10A was next assessed in the testis by immunohistochemistry. In the seminiferous tubules, no specific PDE10A signal was observed in Sertoli cells, spermatogonia or spermatocytes. Although detected in the cytoplasm of elongating spermatids, a strong PDE10A signal was observed in the region of early acrosomal vesicle of round spermatid (Fig 4C and 4F). It was also present in the acrosomal and equatorial region of the elongated spermatids (Fig 4D and 4E, respectively).

### PDE10A is present in bovine spermatozoa

**Fig 5:**
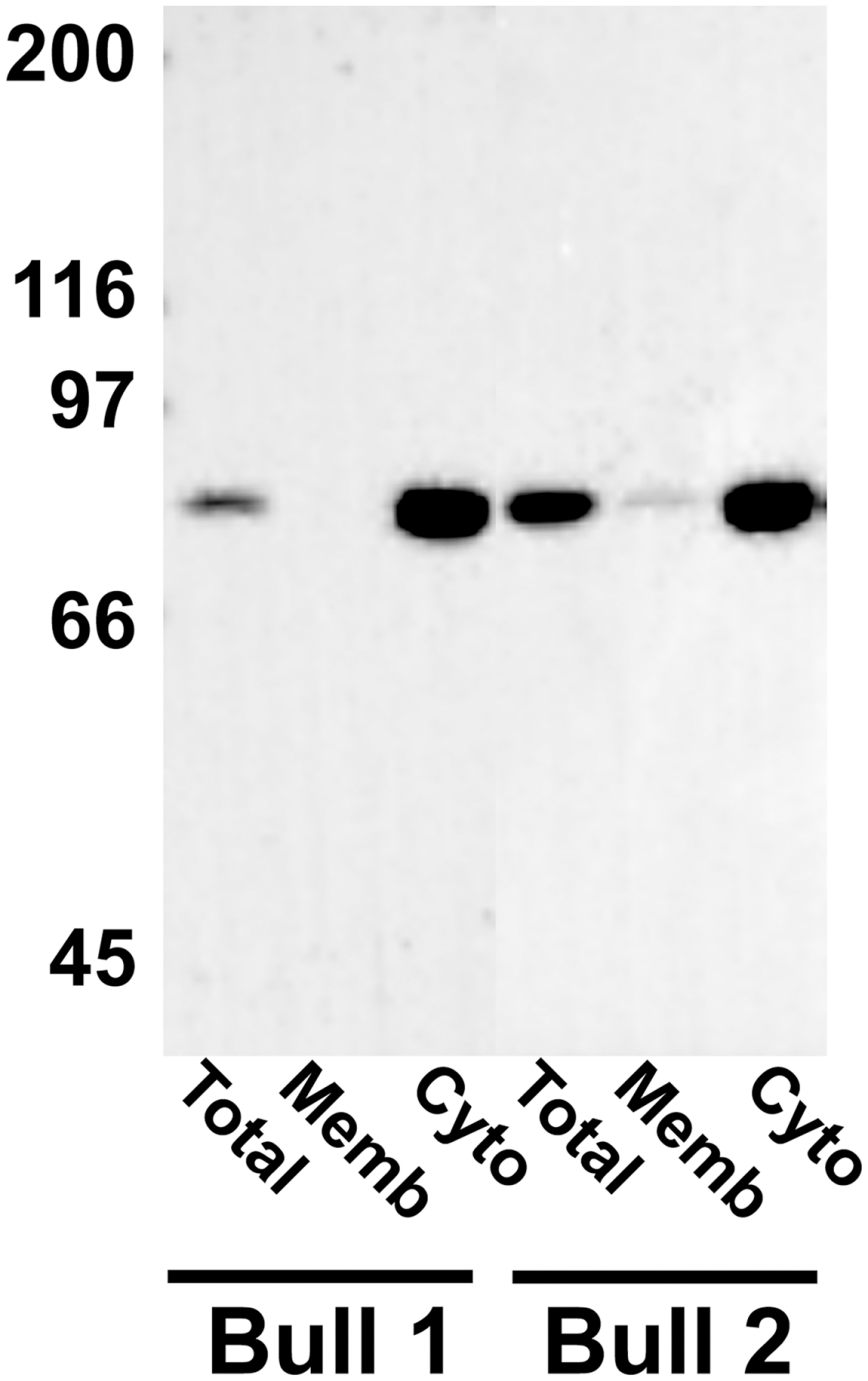
PDE10A in ejaculated bull sperm. Washed spermatozoa were subjected to nitrogen cavitation and the homogenate was centrifuged at low speed and then at high speed to separate raw membranes and cytosolic fractions as described in Materials & Methods. Equal amount of proteins from total cavitated sperm and from the membrane and cytosolic fractions were subjected to electrophoresis, transferred onto a PVDF membrane and probed with a PDE10A monoclonal antibody (clone 1C9). An anti-mouse IgG secondary antibody conjugated to horseradish peroxidase was then used and the positive signal was revealed with ChemiDoc MP Imaging system. Molecular weight standards (kDa) are indicated on the left.

**Fig 6:**
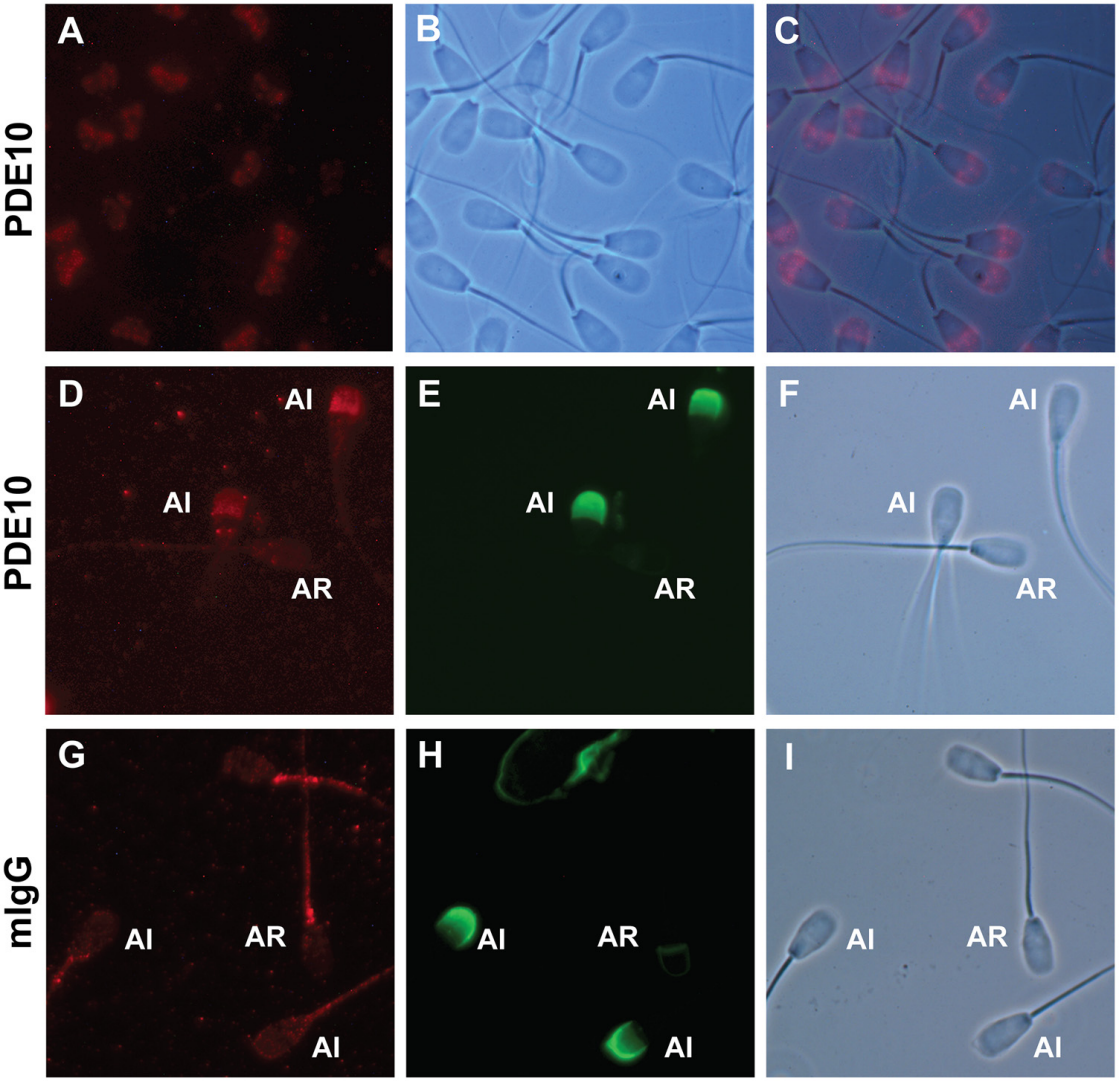
Immunolocalisation of PDE10A in acrosome intact and acrosome reacted ejaculated bull spermatozoa. Washed spermatozoa were fixed with formaldehyde and permeabilized with Triton X-100. They were next incubated overnight with a monoclonal anti-PDE10A antibody (clone 3G9; A-F) or a commercial non-immune mouse IgG at the same concentration (G-I). Panels A, D & G show the positive immunofluorescent signal obtained using a DyLight 594-conjugated secondary antibody; Panels E & H show the presence/absence of the acrosome (PNA-FITC labeling) on spermatozoa from the same field of D & G, respectively. Panels B, F & I are Phase contrast image of the same respective field (A, D & G). Panel C shows the superposition of Panels A & B. Magnification is 1000X. Note that PDE10A is detected in the acrosomal region of acrosome intact (AI) but not of acrosome reacted (AR) sperm (D-F). Some non-specific staining is observed in the midpiece of both AI and AR sperm incubated with non-immune mouse IgG (G-I). Identical PDE10A localization is obtained when the immunodetection experiment is performed in the absence (A) or presence (D) of PNA-FITC, or using a FITC-conjugated secondary antibody (not shown).

**Fig 7:**
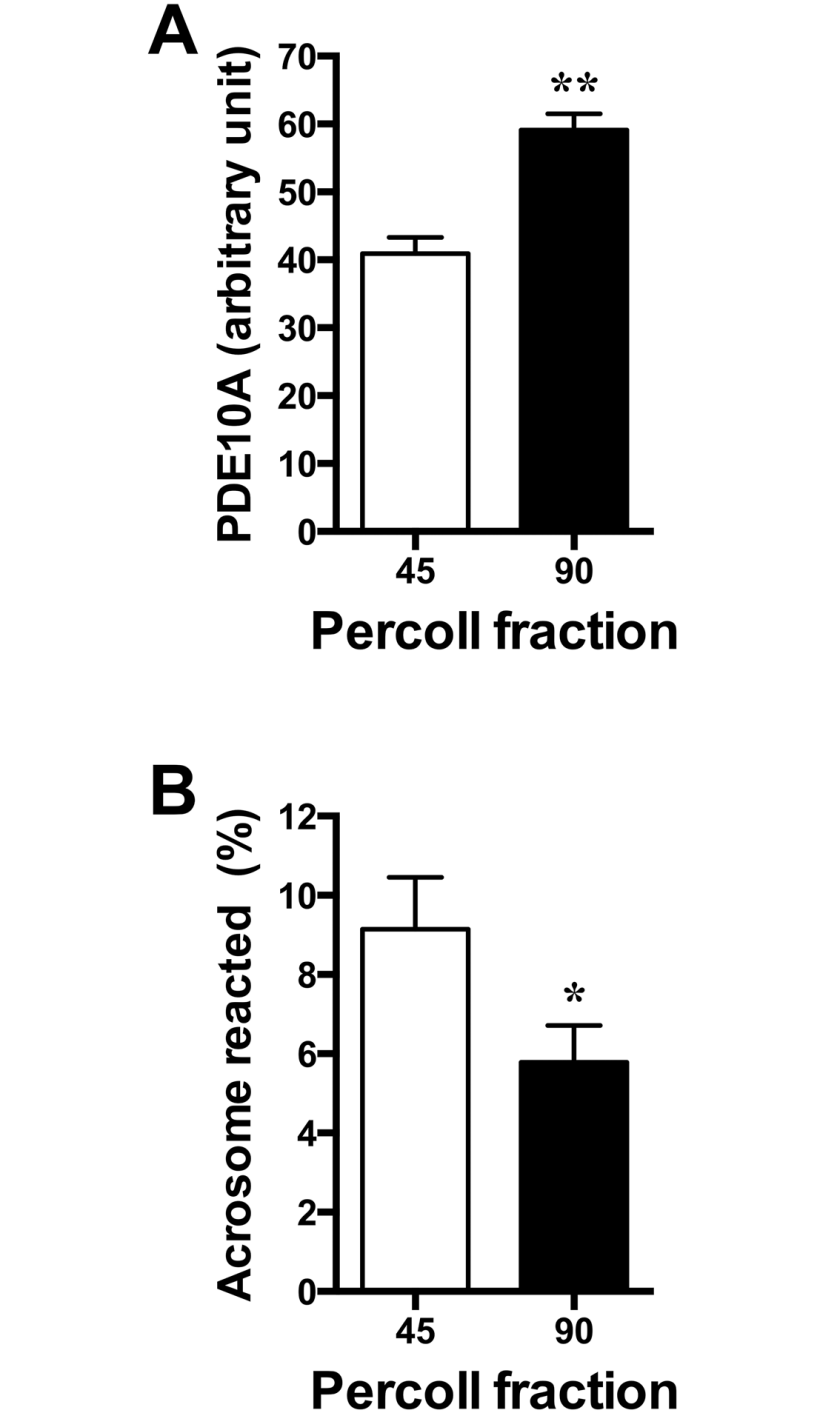
PDE10A in cryopreserved bull spermatozoa. Cryopreserved spermatozoa were thawed and washed by centrifugation through a two-layer (45 and 90%) Percoll density gradient and sperm from the 45/90% interface (45) and within the 90% Percoll fraction (90) were recovered. **A)** Spermatozoa from each fraction were processed for electrophoresis and immunoblotting as described in Fig 5, the intensity of the positive signal was normalized to the intensity of tubulin and, for each bull, expressed as a percentage of the sum of the ratios calculated for the cells recovered at the 45/90% Percoll interface and within the 90% Percoll layer. **B)** Spermatozoa from each fraction were fixed/permeabilized in methanol and then assessed for the presence of the acrosome by PNA-FITC binding. Only sperm with complete absence of acrosomal staining or with staining restricted to the equatorial segment were considered as acrosome reacted (AR). * and ** are significantly different, p <0.05 and p<0.01, respectively from the value obtained in sperm from the 45/90% Percoll interface, (n = 12; paired t test).

Although the transcript variants X3 and X5, encoding PDE10A isoforms X2 and X4, are detected in spermatocytes and spermatids, we sought to determine whether the enzyme is present and where it is localized in mature ejaculated spermatozoa. In western blot analyses, PDE10A was detected in ejaculated bull spermatozoa as a single 81 kDa band (Fig 5). When washed spermatozoa were subjected to nitrogen cavitation followed by centrifugation at different speeds, in the absence of detergent, PDE10A was detected mostly in the supernatant of the high-speed (100,000 x g) centrifugation, suggesting that it is a cytosolic protein or a protein loosely attached to membranes or to any other intracellular compartments. This is further emphasized by its detection, although to a much lower extent, in the raw membrane fraction. Indirect immunofluorescence procedures revealed that PDE10A is localized to the acrosomal region of ejaculated spermatozoa (AI, Fig 6). No PDE10A signal is detected in acrosome reacted sperm (AR, Fig 6) and no change in PDE10A localization was observed when sperm were incubated in the absence or presence of heparin to induce capacitation (data not shown). Furthermore, identical PDE10A localization was observed when the immunofluorescence procedure was done on cauda epididymal sperm or on cryopreserved sperm (data not shown). When cryopreserved sperm were thawed and washed by centrifugation through a two layer (45 and 90%) Percoll density gradient, a higher level of PDE10A protein was detected in the 90% Percoll fraction, which contains the highly motile sperm population [54] and the lowest level of spermatozoa with damaged or reacted acrosome (Fig 7). From these latter results, the negative correlation between PDE10A levels and acrosome reacted sperm percentage almost reach statistical significance (Pearson’s correlation coefficient r = -0.38; p = 0.06).

**Fig 8:**
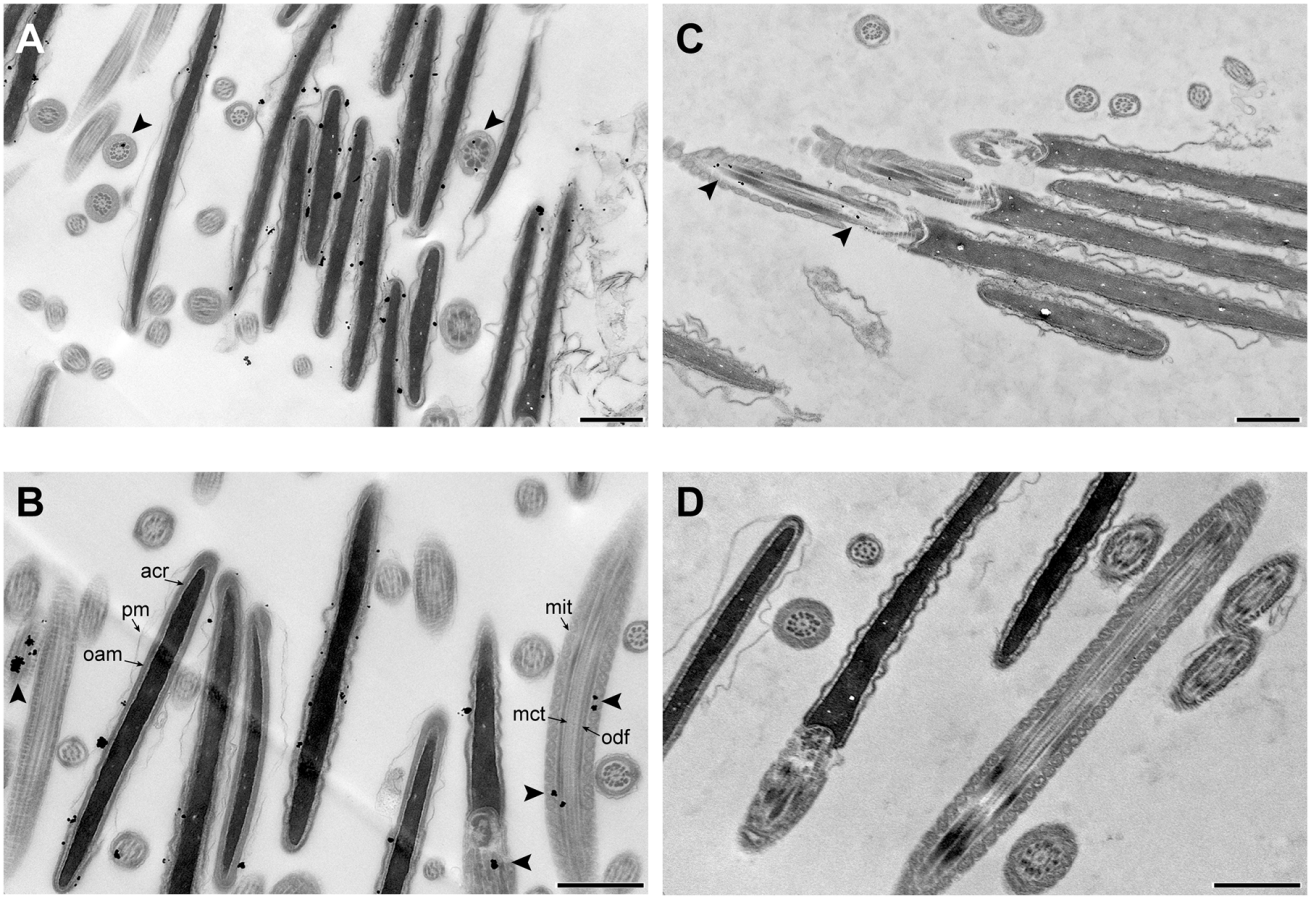
Detection of bull sperm PDE10A at the electron microscopic level. Freshly ejaculated bull spermatozoa were washed, fixed with acrolein and pelleted in agarose. Sections of 50 μm were obtained and processed for immunodetection as described in Materials & Methods using monoclonal anti PDE10A antibody (A & B) or non-immune commercial mouse IgG (C & D). Sections were then post-fixed, embedded, and ultrathin sections were prepared and observed by transmission electron microscopy. Note that there is some non-specific signal in the midpiece region that is observed with both the PDE10A antibody and the non-immune IgG (arrowheads in A, B & C). Photographs were taken with magnification of 6800 X (A & C) and 9300 X (B & C). Acrosomal matrix (acr), plasma membrane (pm), and outer acrosomal membrane (oam; lining the acrosomal matrix) are indicated in the sperm head; mitochondria (mit), microtubules (mit) and outer dense fibers (odf) are indicated in the sperm midpiece. In all the panels, the bar scale represents 1 μm.

When the localization of PDE10A was assessed at the electron microscopic level to determine the precise location of this enzyme in the acrosomal area, positive signal was associated with the outer acrosomal membrane or within the acrosomal matrix, immediately underneath the outer acrosomal membrane (Fig 8). This result further supports the absence of PDE10A signal in acrosome reacted sperm as these cells lose their plasma membrane overlying the acrosome as well as the outer acrosomal membrane and the acrosomal matrix during acrosome reaction.

**Fig 9:**
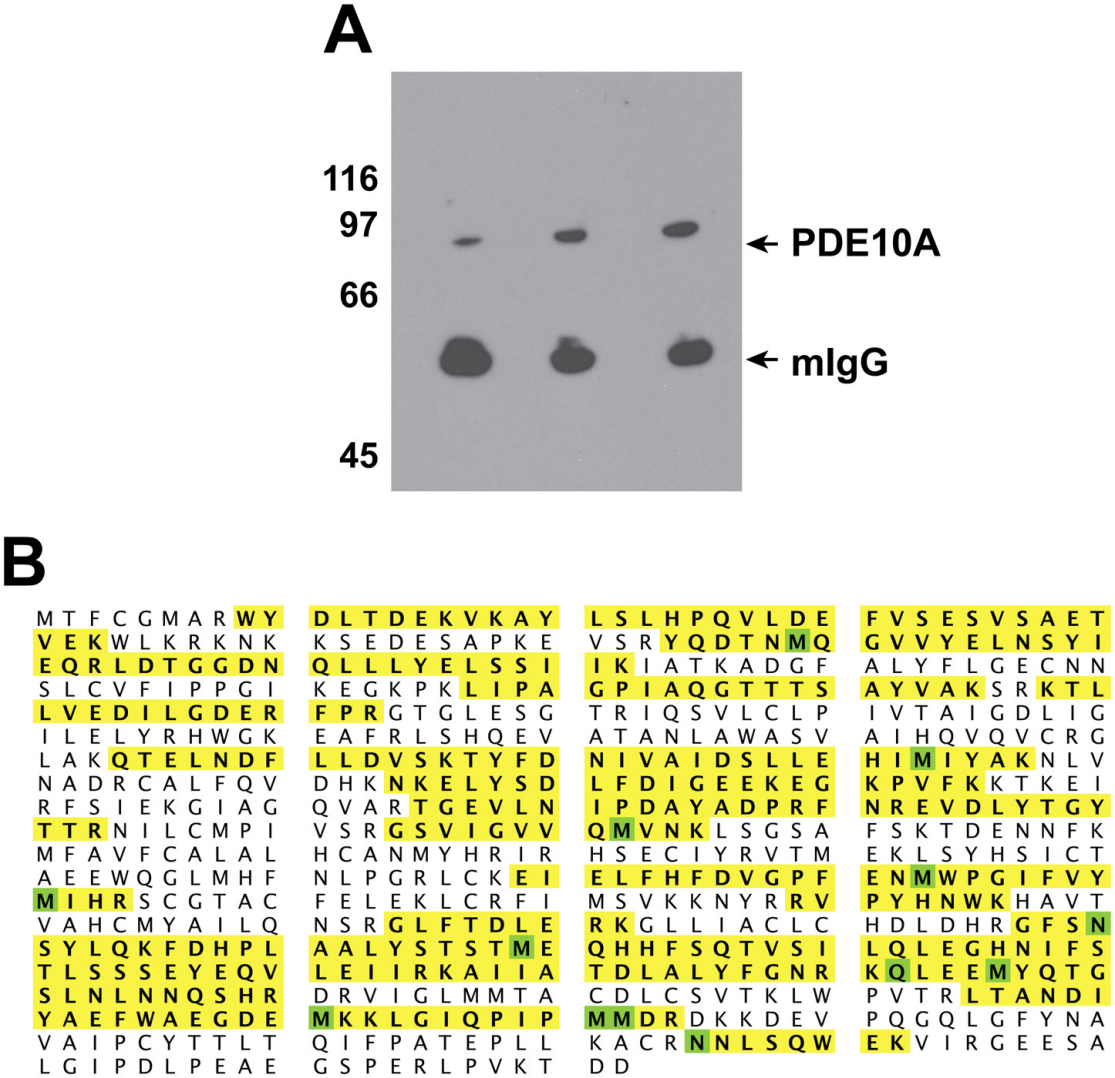
Immunoprecipitation and identification of bull sperm PDE10A. A) Ejaculated bull spermatozoa were processed for Immunoprecipitation as described in Materials & Methods using a PDE10A monoclonal antibody and protein G-coupled sepharose. The immune-complex on the beads was next solubilized with sample buffer, subjected to electrophoresis and transferred onto PVDF membrane. Proteins were next probed with a monoclonal anti-PDE10A antibody and revealed with a secondary antibody conjugated to horseradish peroxidase, enhanced chemiluminescence, and film exposure. Molecular weight standards (kDa) are indicated on the left. B) Coverage of the PDE10A isoform X4 amino acid sequence by the trypsin generated peptides identified by LC-MS/MS. Sequence sections highlighted in yellow matched the peptides identified. Amino acids highlighted in green show variable post-translational modifications (Oxidation of M, Deamination of Q or N).

Since none of the antibodies used could discriminate between the different PDE10A isoforms, attempts were made to identify specific sperm PDE10A isoforms by LC-MS/MS. Taking advantage of the success of immunoprecipitation of sperm PDE10A (Fig 9A), immune-complexes attached to the sepharose beads coupled to protein G were sent for analysis to the Proteomic platform facility of our research center. A total of 8 samples were sent for identification. For each sample, between 8 and 36 (average 19) unique peptides specific for PDE10A were identified, which corresponded to a sequence coverage ranging from 14 to 49% (average 29%). In 7 out of the 8 samples, a peptide specific to the N-terminal portion of isoform X4 was identified, which strongly suggests that isoform X4 (transcript variant X5) is the major PDE10A isoform present in bull sperm. The sequence coverage of the sample with 36 identified peptides is shown in Fig 9B. However, our results do not exclude the possibility that isoform X2 (transcript variant X3) is also present in mature sperm but no peptide specific to X2 N-terminal domain was identified in any of our samples processed for identification.

## Discussion

In the bovine specie, the gene encoding *PDE10A* spans over 353 kb and is found on chromosome 9 (http://www.ncbi.nlm.nih.gov/gene?cmd=retrieve&dopt=full_report&list_uids=506061), whereas in humans, it spans over 200 kb on chromosome 6 [44]. In human, Northern blot analyses revealed that the size of the transcript encoding *PDE10A* is 10 kb in numerous tissues although another transcript of 4.0 kb is present in the testis [38]. When adding the number of nucleotides obtained and sequenced in our 5’- and the 3’-RACE experiments to the number of nucleotides present between the gene specific primers that we used for these 2 experiments, a transcript up to 3.9 kb is obtained, which is highly similar to the size of the human [38] and rat [39] testicular *PDE10A* transcripts detected by Northern blot.

**Fig 10:**
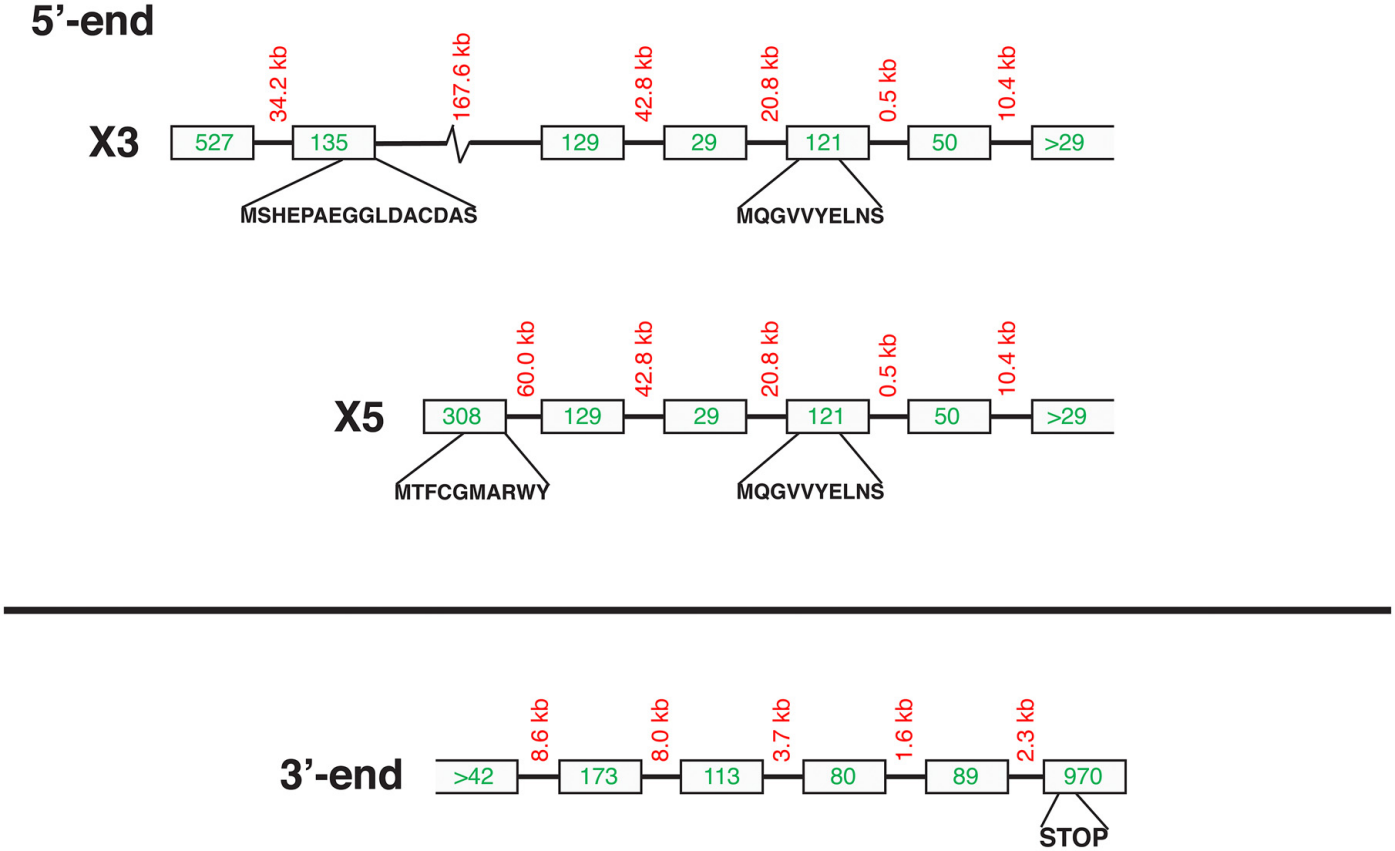
Putative intron-exon distribution in the 5'- and 3'-ends of bovine *PDE10A* gene. Intron-exon distribution deduced from the sequences obtained in our 5'-RLM (upper part) and 3'-RACE (lower part) experiments is presented. The numbers in the boxes refer to the number of nucleotides in the putative exons. The length of the introns is also indicated between the boxes. The position of the start of the ORFs in the variant X3 (isoform X2; **MSHEPAEGGLDACDAS**), the variant X5 (isoform X4; **MTFCGMARWY**), as well as the initial Methionine residue deduced from the different clones obtained in our 5’-RLM experiments that were similar to the variant X5 although with deletions in the 5’-UTR (**MQGVVYELNS**), are indicated. This ‘alternate’ start codon lies in the portion of the protein that is shared by all predicted PDE10A isoforms.

Different testicular PDE10A isoforms resulting from 3 unique N-termini and 2 C-termini, resulting from alternative splicing have been reported in humans [44]. A total of six putative PDE10A isoforms can be obtained from the different combinations of N- and C-termini. In our 5’-RACE study, we obtained 5 different 5’ ends that would encode *PDE10A* variant X3 (NCBI accession number XM_005211114), variant X5 (XM_005211116) as well as variant X5 with slight variations. The deduced amino acid sequence would reveal 3 different PDE10A N-termini (Fig 10): **MQGVVYELNS**, which is common to all PDE10A isoforms, **MTFCGMARWY**, specific to isoform X4, and **MSHEPAEGGLDACDAS,** which is the N-terminus of isoforms X2 and X3. The transcript variants encoding these 2 isoforms differ at the nucleotide level in the 5’-UTR but, in our experiments, the sequences obtained were similar to the one encoding the variant X3.

Previous reports have shown that PDE10A is a cytosolic enzyme with the exception of PDE10A2, which is present in the membrane fraction [55]. It has been demonstrated that PDE10A2 association with the membrane is under the regulation of cAMP/PKA-dependent phosphorylation. The threonine residue (Thr^16^) in the N-terminal region of PDE10A2 is a substrate for cAMP/PKA-dependent phosphorylation and needs to be dephosphorylated to allow the association of PDE10A2 with membranes [55]. To prevent the association of PDE10A2 with membranes, phosphorylation of Thr^16^ would occur, interfering with palmitoylation of Cys^11^, which normally promotes membrane association [56]. In the present study, from the deduced amino acid sequences of the X2 and X4 isoforms most likely present in testicular germ cells and mature spermatozoa, only the X4 has a Thr residue (Thr^2^) which, according to the proteomic prediction tools (GPS, http://gps.biocuckoo.org), can be phosphorylated although in a cAMP/PKA independent manner. In addition, from the palmitoylation prediction tool (GPS, http://gps.biocuckoo.org), Cys^4^ of the bull sperm PDE10A isoform X4 is a putative candidate for palmitoylation, unlike isoform X2 which does not possess a Cys residue putatively palmitoylated in its N-terminal region. Since our results clearly show that sperm PDE10A is present mostly in the cytosolic fraction (Fig 5), this would suggest that the enzyme is generally phosphorylated on Thr^2^, or that phosphorylation of the residue does not regulate membrane/cytosol distribution of PDE10A. The absence of palmitoylated Cys^4^ would also explain the presence of PDE10A in the sperm cytosolic fraction. Nonetheless, post-translational modification, phosphorylation and palmitoylation, of these two residues remain to be elucidated.

PDE10A has been mostly studied in the brain as it is associated with diseases such as the neurodegenerative genetic Huntington’s disease [41], psychosis [42], and schizophrenia [43]. In the testis, however, very little is known. Different cell types are present in the testes, such as interstitial androgen-producing Leydig cells, as well as cells within the seminiferous tubule: the Sertoli cells and the germ cells present at different stage of spermatogenesis and spermiogenesis. Our immunohistochemical studies on testis sections show that PDE10A is absent from the somatic cells. On the other hand, although the transcript encoding PDE10A is present in both primary spermatocytes and spermatids, the enzyme is detected only in these latter haploid cells. A strong PDE10A expression is observed in the regions of early acrosomal vesicle, equatorial segment, and developed acrosome of testicular spermatozoa. This is in perfect agreement with its detection in the acrosomal region of ejaculated sperm. The absence of a PDE10A signal in acrosome reacted sperm strongly suggests that this enzyme is not associated with the inner acrosomal membrane, which remains associated with the sperm head following the acrosome reaction. This is further supported by our immunoelectron microscopy experiments showing that it is associated with the outer acrosomal membrane and/or immediately underneath, in the acrosomal matrix.

Previous studies using inhibitors have suggested that PDE4 is involved in sperm motility, which corroborates with its localization in the flagellum, most likely associated with AKAP3 [31, 33]. On the other hand, PDE1 would be involved in capacitation-associated modifications that occur in the acrosomal/head region of spermatozoa [31, 32], which is in agreement with its localization in the equatorial region of the sperm head [30]. Although PDE3 is detected in the postacrosomal region of the human sperm head [30], no specific role for this PDE in sperm function has been demonstrated so far. Therefore, because of the presence of PDE10A in the acrosomal region, it is tempting to hypothesize that it is somehow involved in the control of sperm acrosomal exocytosis. In fact, this appears to be the case as an increase in spontaneous acrosome reaction was observed when sperm are incubated with the specific PDE10 inhibitor MP10 [57] (data not shown, manuscript in preparation), while no effect on sperm motility was observed (63.0% ± 7.5 vs 66.3% ± 4.8 for control vs MP10-treated sperm). We previously showed that sperm cAMP concentration is correlated with the membrane-binding pattern of chlortetracycline (CTC, pattern B) that is generally observed in capacitated sperm [58, 59]. Similarly, sperm cAMP levels are correlated with the amount of Ca^2+^ that is released from internal stores, such as the acrosome, upon thapsigargin challenge [58, 59]. Since PDE10A is present in the acrosomal region of the head, it might be involved in the regulation of acrosomal Ca^2+^ storage/mobilization through localized regulation of cAMP levels. PDE10A might therefore be the PDE responsible for the higher levels of thapsigargin-mobilized Ca^2+^ measured in sperm capacitated in the presence of the general PDE inhibitor IBMX [58, 59]. Future studies are required to determine whether PDE10A is involved in the storage of sperm intracellular Ca^2+^.

This study is the first to clearly demonstrate the presence of PDE10A in spermatozoa and to identify the major isoform present in this cell type. As expected from the reported high level of *PDE10A* transcript in the testis, the enzyme is present in this organ. PDE10A is detected in the seminiferous tubules in spermatids, with a strong signal at the developing acrosome level. In mature spermatozoa, PDE10A is localized in the acrosomal region of the head and our results obtained with a specific inhibitor suggest that it is involved in sperm capacitation or acrosome reaction. Results from 5’-RACE, 3’-RACE and PCR experiments revealed that testicular cells, including primary spermatocytes and spermatids, express the transcript variants X3 and X5. Mass spectrometry analysis of immunoprecipitated PDE10A demonstrated that X4 is the most abundant isoform present in ejaculated bull sperm. Further studies are required to determine the role, the activity, and the regulation of PDE10A in sperm function.

## Supporting Information

S1 Fig Sequence alignment of one of the clones obtained in our 5’-RLM experiment with the sequence of *PDE10A* transcript variant X3. (PDF)

S2 Fig Sequence alignment of one of the clones obtained in our 5’-RLM experiment with the sequence of *PDE10A* transcript variant X5. (PDF)

S3 Fig Sequence alignment of one of the clones obtained in our 5’-RLM experiment with the sequence of *PDE10A* transcript variant X5. The clone lacks a stretch of 5 nucleotides (GGTAT) that causes a shift in the position of the starting Met residue and, therefore, of the open reading frame.
(PDF)

S4 Fig Sequence alignment of one of the clones obtained in our 5’-RLM experiment with the sequence of *PDE10A* transcript variant X5. Clones’ sequence from nucleotide 9–155 is present in the genomic sequence but might come from mis-spliced RNA. The stretch from nucleotides 9 to 180 of transcript variant X5 is missing from these clones.
(PDF)

S5 Fig Sequence alignment of one of the clones obtained in our 5’-RLM experiment with the sequence of *PDE10A* transcript variant X5. Clones’ sequence from nucleotide 1–102 is present in the genomic sequence but might come from mis-spliced RNA, as for the sequence from nucleotide 608–668. Note the absence of 5 nucleotides (GGTAT; nucleotides 176–180 of transcript variant X5) that causes a shift in the position of the starting Met residue and, therefore, of the open reading frame.
(PDF)
