# Supplementary material for: Identification and Localization of the Cyclic Nucleotide Phosphodiesterase 10A in Bovine Testis and Mature Spermatozoa
Source: PLoS One. 2016 Aug 22;11(8):e0161035. doi: 10.1371/journal.pone.0161035 (PMC4993467; doi:10.1371/journal.pone.0161035)
Supplement: S1 Fig — (PDF) [file pone.0161035.s001.pdf]

```
clone           AAGTGCTTCCGGTCCATCCGCTCTGTTCGCCGTGGCCCAGGGGTGGACGTGGACGTGAGG 60
X3              -----------------CCGCTCTGTTCGCCGTGGCCCAGGGGTGGACGTGGACGTGAGG 43
                                 *******************************************

clone           CCTCACAAGGCCACCCGCAACTCCTCGGAGGTCCCTGCACCGTCTCTGCCGTCCTTGTTT 120
X3              CCTCACAAGGCCACCCGCAACTCCTCGGAGGTCCCTGCACCGTCTCTGCCGTCCTTGTTT 103
                ************************************************************

clone           CCCTCAACTGCAGTCTTCCGAATCCTTGAAAAGTTCTCAGGACTCCAGTCCTTTCTTGCT 180
X3              CCCTCAACTGCAGTCTTCCGAATCCTTGAAAAGTTCTCAGGACTCCAGTCCTTTCTTGCT 163
                ************************************************************

clone           TTTCAGTTGGCCATTTTGACCACTTCAATTTCAGAACTTAAATTCGGATAATTCCTCCTA 240
X3              TTTCAGTTGGCCATTTTGACCACTTCAATTTCAGAACTTAAATTCGGATAATTCCTCCTA 223
                ************************************************************

clone           ATCGTTCCAATGCGCTCGAGGTCTCTGCACGAACTGCAGCTCTCATCCTCCACCGACTCA 300
X3              ATCGTTCCAATGCGCTCGAGGTCTCTGCACGAACTGCAGCTCTCATCCTCCACCGACTCA 283
                ************************************************************

clone           GGCAGCGTCTTTCCCTTCACTGAACAAAAGCCTGTTTCCGCGGCTTCCAACTCTGCGCAG 360
X3              GGCAGCGTCTTTCCCTTCACTGAACAAAAGCCTGTTTCCGCGGCTTCCAACTCTGCGCAG 343
                ************************************************************

clone           TGCCCACACGCCTGCCACCCACCCGTGCCTTCCTTCTGTCAAGTCGACTCCCTCTTCACA 420
X3              TGCCCACACGCCTGCCACCCACCCGTGCCTTCCTTCTGTCAAGTCGACTCCCTCTTCACA 403
                ************************************************************

clone           TGGCTCCCGCAGAAGCCTGTCAGTCAAGGAGGACCAGGAAGCCAGATCCCAGCAACCCCA 480
X3              TGGCTCCCGCAGAAGCCTGTCAGTCAAGGAGGACCAGGAAGCCAGATCCCAGCAACCCCA 463
                ************************************************************

clone           CGTCTCACACACTCCCGCTAAACACCACACAGAAAACTTCAGGAAATCCAGCTGCCTCCC 540
X3              CGTCTCACACACTCCCGCTAAACACCACACAGAAAACTTCAGGAAATCCAGCTGCCTCCC 523
                ************************************************************

clone           TTTCACACTGAAGAGCGGGAGCAGAGGACCCGCTTTGCTGACCCTGAGAAGCAGGACAGA 600
X3              TTTCACACTGAAGAGCGGGAGCAGAGGACCCGCTTTGCTGACCCTGAGAAGCAGGACAGA 583
                ************************************************************

clone           CCCCCAGGAGCAGATGAGCCATGAGCCAGCGGAAGGCGGCCTGGACGCCTGCGATGCCTC 660
X3              CCCCCAGGAGCAGATGAGCCATGAGCCAGCGGAAGGCGGCCTGGACGCCTGCGATGCCTC 643
                ************************************************************

clone           AGATTTGACAGATGAAAAAGTGAAGGCCTATCTTTCTCTCCACCCTCAGGTTTTAGATGA 720
X3              AGATTTGACAGATGAAAAAGTGAAGGCCTATCTTTCTCTCCACCCTCAGGTTTTAGATGA 703
                ************************************************************

clone           ATTTGTGTCTGAAAGCGTTAGTGCAGAGACTGTAGAAAAATGGCTGAAGCGGAAAAACAA 780
X3              ATTTGTGTCTGAAAGCGTTAGTGCAGAGACTGTAGAAAAATGGCTGAAGCGGAAAAACAA 763
                ************************************************************

clone           GAAGTCAGAAGATGAATCAGCTCCTAAGGAAGTCAGCAGGTATCAAGATACAAATATGCA 840
X3              GAAGTCAGAAGATGAATCAGCTCCTAAGGAAGTCAGCAGGTATCAAGATACAAATATGCA 823
                ************************************************************

clone           AGGAGTTGTGTATGAACTAAATAGCTATATAGAACAGCGGTTGGATACAGGAGGAGACAA 900
X3              AGGAGTTGTGTATGAACTAAATAGCTATATAGAACAGCGGTTGGATACAGGAGGAGACAA 883
                ************************************************************

clone           CCAGCTACTCCTCTATGAACTGAGCAGCATCATCAAAATCGCCACAAAAGCTGACGGATT 960
X3              CCAGCTACTCCTCTATGAACTGAGCAGCATCATCAAAATCGCCACAAAAGCTGACGGATT 943
                ************************************************************

clone           TGCACTGTATTTCCTTGGAGAGTGCAATAACAGCCTTTGTGTGTTTATCCCGCCTGGAAT 1020
X3              TGCACTGTATTTCCTTGGAGAGTGCAATAACAGCCTTTGTGTGTTTATCCCGCCTGGAAT 1003
                ************************************************************
```
