# Supplementary material for: Identification and Localization of the Cyclic Nucleotide Phosphodiesterase 10A in Bovine Testis and Mature Spermatozoa
Source: PLoS One. 2016 Aug 22;11(8):e0161035. doi: 10.1371/journal.pone.0161035 (PMC4993467; doi:10.1371/journal.pone.0161035)
Supplement: S2 Fig — (PDF) [file pone.0161035.s002.pdf]

```
clone           GGAATCTGTTGGAGGGCGGTTGGTCAGTCCTCGGCCTGAGAGAGCTGGGCAGCGGGAGAC 60
X5

clone           TCTGCCGGTGTGTCTGCTTGGACTCCGGGTGGAGAGGAGGCCGTCTGAGGACTCGTGAGC 120
X5

clone           AAGCCCTTGTCCCCAGGAAAGCCCGCAGGCCGGATGTCAGCCCCGGGAGTCAGGCTGCCC 180
X5                     TGTCCCCAGGAAAGCCCGCAGGCCGGATGTCAGCCCCGGGAGTCAGGCTGCCC 53
                       *****************************************************

clone           GAGAGGCCGGGCGGGCCGGAGGGGCCAGCTTAGCAGCGCTGTGCGGTCGACCAGAGAAGC 240
X5              GAGAGGCCGGGCGGGCCGGAGGGGCCAGCTTAGCAGCGCTGTGCGGTCGACCAGAGAAGC 113
                ************************************************************

clone           CCTCCTGCTTCCTTCTGCCTGCGAACCCCGGCAGCTAATGACTTTCTGTGGGATGGCAAG 300
X5              CCTCCTGCTTCCTTCTGCCTGCGAACCCCGGCAGCTAATGACTTTCTGTGGGATGGCAAG 173
                ************************************************************

clone           ATGGTATGATTTGACAGATGAAAAAGTGAAGGCCTATCTTTCTCTCCACCCTCAGGTTTT 360
X5              ATGGTATGATTTGACAGATGAAAAAGTGAAGGCCTATCTTTCTCTCCACCCTCAGGTTTT 233
                ************************************************************

clone           AGATGAATTTGTGTCTGAAAGCGTTAGTGCAGAGACTGTAGAAAAATGGCTGAAGCGGAA 420
X5              AGATGAATTTGTGTCTGAAAGCGTTAGTGCAGAGACTGTAGAAAAATGGCTGAAGCGGAA 293
                ************************************************************

clone           AAACAAGAAGTCAGAAGATGAATCAGCTCCTAAGGAAGTCAGCAGGTATCAAGATACAAA 480
X5              AAACAAGAAGTCAGAAGATGAATCAGCTCCTAAGGAAGTCAGCAGGTATCAAGATACAAA 353
                ************************************************************

clone           TATGCAAGGAGTTGTGTATGAACTAAATAGCTATATAGAACAGCGGTTGGATACAGGAGG 540
X5              TATGCAAGGAGTTGTGTATGAACTAAATAGCTATATAGAACAGCGGTTGGATACAGGAGG 413
                ************************************************************

clone           AGACAACCAGCTACTCCTCTATGAACTGAGCAGCATCATCAAAATCGCCACAAAAGCTGA 600
X5              AGACAACCAGCTACTCCTCTATGAACTGAGCAGCATCATCAAAATCGCCACAAAAGCTGA 473
                ************************************************************

clone           CGGATTTGCACTGTATTTCCTTGGAGAGTGCAATAACAGCCTTTGTGTGTTTATCCCGCC 660
X5              CGGATTTGCACTGTATTTCCTTGGAGAGTGCAATAACAGCCTTTGTGTGTTTATCCCGCC 533
                ************************************************************

clone           TGGAAT 666
X5              TGGAAT 539
                ******
```
