# Supplementary material for: Identification and Localization of the Cyclic Nucleotide Phosphodiesterase 10A in Bovine Testis and Mature Spermatozoa
Source: PLoS One. 2016 Aug 22;11(8):e0161035. doi: 10.1371/journal.pone.0161035 (PMC4993467; doi:10.1371/journal.pone.0161035)
Supplement: S3 Fig — The clone lacks a stretch of 5 nucleotides (GGTAT) that causes a shift in the position of the starting Met residue and, therefore, of the open reading frame. (PDF) [file pone.0161035.s003.pdf]

```
clone           CCCCAGCCCATGGCAGTTGGAATCTGTTGGAGGGCGGTTGGTCAGTCCTCGGCCTGAGAG 60
X5              

clone           AGCTGGGCAGCGGGAGACTCTGCCGGTGTGTCTGCTTGGACTCCGGGTGGAGAGGAGGCC 120
X5              

clone           GTCTGAGGACTCGTGAGCAAGCCCTTGTCCCCAGGAAAGCCCGCAGGCCGGATGTCAGCC 180
X5                                       TGTCCCCAGGAAAGCCCGCAGGCCGGATGTCAGCC 35

clone           CCGGGAGTCAGGCTGCCCGAGAGGCCGGGCGGGCCGGAGGGGCCAGCTTAGCAGCGCTGT 240
X5              CCGGGAGTCAGGCTGCCCGAGAGGCCGGGCGGGCCGGAGGGGCCAGCTTAGCAGCGCTGT 95
                ************************************************************

clone           GCGGTCGACCAGAGAAGCCCTCCTGCTTCCTTCTGCCTGCGAACCCCGGCAGCTAATGAC 300
X5              GCGGTCGACCAGAGAAGCCCTCCTGCTTCCTTCTGCCTGCGAACCCCGGCAGCTAATGAC 155
                ************************************************************

clone           TTTCTGTGGGATGGCAAGAT-----GATTTGACAGATGAAAAAGTGAAGGCCTATCTTTC 355
X5              TTTCTGTGGGATGGCAAGATGGTATGATTTGACAGATGAAAAAGTGAAGGCCTATCTTTC 215
                ********************     ***********************************

clone           TCTCCACCCTCAGGTTTTAGATGAATTTGTGTCTGAAAGCGTTAGTGCAGAGACTGTAGA 415
X5              TCTCCACCCTCAGGTTTTAGATGAATTTGTGTCTGAAAGCGTTAGTGCAGAGACTGTAGA 275
                ************************************************************

clone           AAAATGGCTGAAGCGGAAAAACAAGAAGTCAGAAGATGAATCAGCTCCTAAGGAAGTCAG 475
X5              AAAATGGCTGAAGCGGAAAAACAAGAAGTCAGAAGATGAATCAGCTCCTAAGGAAGTCAG 335
                ************************************************************

clone           CAGGTATCAAGATACAAATATGCAAGGAGTTGTGTATGAACTAAATAGCTATATAGAACA 535
X5              CAGGTATCAAGATACAAATATGCAAGGAGTTGTGTATGAACTAAATAGCTATATAGAACA 395
                ************************************************************

clone           GCGGTTGGATACAGGAGGAGACAACCAGCTACTCCTCTATGAACTGAGCAGCATCATCAA 595
X5              GCGGTTGGATACAGGAGGAGACAACCAGCTACTCCTCTATGAACTGAGCAGCATCATCAA 455
                ************************************************************

clone           AATCGCCACAAAAGCTGACGGATTTGCACTGTATTTCCTTGGAGAGTGCAATAACAGCCT 655
X5              AATCGCCACAAAAGCTGACGGATTTGCACTGTATTTCCTTGGAGAGTGCAATAACAGCCT 515
                ************************************************************

clone           TTGTGTGTTTATCCCGCCTGGAAT 679
X5              TTGTGTGTTTATCCCGCCTGGAAT 539
                ************************
```
