# Supplementary material for: Identification and Localization of the Cyclic Nucleotide Phosphodiesterase 10A in Bovine Testis and Mature Spermatozoa
Source: PLoS One. 2016 Aug 22;11(8):e0161035. doi: 10.1371/journal.pone.0161035 (PMC4993467; doi:10.1371/journal.pone.0161035)
Supplement: S4 Fig — Clones’ sequence from nucleotide 9–155 is present in the genomic sequence but might come from mis-spliced RNA. The stretch from nucleotides 9 to 180 of transcript variant X5 is missing from these clones. (PDF) [file pone.0161035.s004.pdf]

```
clone           GTCCCCAGCCCATGGCAGTTGGAATCTGTTGGAGGGCGGTTGGTCAGTCCTCGGCCTGAG 60
X5              GTCCCCA----------------------------------------------------- 8
                *******

clone           AGAGCTGGGCAGCGGGAGACTCTGCCGGTGTGTCTGCTTGGACTCCGGGTGGAGAGGAGG 120
X5              ------------------------------------------------------------


clone           CCGTCTGAGGACTCGTGAGCAAGCCCTTGTCCCCAGATTTGACAGATGAAAAAGTGAAGG 180
X5              -----------------------------------GATTTGACAGATGAAAAAGTGAAGG 205
                                                   *************************

clone           CCTATCTTTCTCTCCACCCTCAGGTTTTAGATGAATTTGTGTCTGAAAGCGTTAGTGCAG 240
X5              CCTATCTTTCTCTCCACCCTCAGGTTTTAGATGAATTTGTGTCTGAAAGCGTTAGTGCAG 265
                ************************************************************

clone           AGACTGTAGAAAAAATGGCTGAAGCGGAAAAACAAGAAGTCAGAAGATGAATCAGCTCCTA 300
X5              AGACTGTAGAAAAAATGGCTGAAGCGGAAAAACAAGAAGTCAGAAGATGAATCAGCTCCTA 325
                ************************************************************

clone           AGGAAGTCAGCAGGTATCAAGATACAAATATGCAAGGAGTTGTGTATGAACTAAATAGCT 360
X5              AGGAAGTCAGCAGGTATCAAGATACAAATATGCAAGGAGTTGTGTATGAACTAAATAGCT 385
                ************************************************************

clone           ATATAGAACAGCGGTTGGATACAGGAGGAGACAACCAGCTACTCCTCTATGAACTGAGCA 420
X5              ATATAGAACAGCGGTTGGATACAGGAGGAGACAACCAGCTACTCCTCTATGAACTGAGCA 445
                ************************************************************

clone           GCATCATCAAAATCGCCACAAAAGCTGACGGATTTGCACTGTATTTCCTTGGAGAGTGCA 480
X5              GCATCATCAAAATCGCCACAAAAGCTGACGGATTTGCACTGTATTTCCTTGGAGAGTGCA 505
                ************************************************************

clone           ATAACAGCCTTTGTGTGTTTATCCCGCCTGGAAT 514
X5              ATAACAGCCTTTGTGTGTTTATCCCGCCTGGAAT 539
                **********************************
```
