# Supplementary material for: Identification and Localization of the Cyclic Nucleotide Phosphodiesterase 10A in Bovine Testis and Mature Spermatozoa
Source: PLoS One. 2016 Aug 22;11(8):e0161035. doi: 10.1371/journal.pone.0161035 (PMC4993467; doi:10.1371/journal.pone.0161035)
Supplement: S5 Fig — Clones’ sequence from nucleotide 1–102 is present in the genomic sequence but might come from mis-spliced RNA, as for the sequence from nucleotide 608–668. Note the absence of 5 nucleotides (GGTAT; nucleotides 176–180 of transcript variant X5) that causes a shift in the position of the starting Met residue and, therefore, of the open reading frame. (PDF) [file pone.0161035.s005.pdf]

```
clone           AGTCCTCGGCCTGAGAGAGCTGGGCAGCGGGAGACTCTGCCGGTGTGTCTGCTTGGACTC 60
X5              ------------------------------------------------------------

clone           CGGGTGGAGAGGAGGCCGTCTGAGGACTCGTGAGCAAGCCCTTGTCCCCAGGAAAGCCCG 120
X5              ------------------------------------------TGTCCCCAGGAAAGCCCG 18
                                                          ******************

clone           CAGGCCGGATGTCAGCCCCGGGAGTCAGGCTGCCCGAGAGGCCGGGCGGGCCGGAGGGGC 180
X5              CAGGCCGGATGTCAGCCCCGGGAGTCAGGCTGCCCGAGAGGCCGGGCGGGCCGGAGGGGC 78
                ************************************************************

clone           CAGCTTAGCAGCGCTGTGCGGTCGACCAGAGAAGCCCTCCTGCTTCCTTCTGCCTGCGAA 240
X5              CAGCTTAGCAGCGCTGTGCGGTCGACCAGAGAAGCCCTCCTGCTTCCTTCTGCCTGCGAA 138
                ************************************************************

clone           CCCCGGCAGCTAATGACTTTCTGTGGGATGGCAAGAT-----GATTTGACAGATGAAAAA 295
X5              CCCCGGCAGCTAATGACTTTCTGTGGGATGGCAAGATGGTATGATTTGACAGATGAAAAA 198
                *************************************     ******************

clone           GTGAAGGCCTATCTTTCTCTCCACCCTCAGGTTTTAGATGAATTTGTGTCTGAAAGCGTT 355
X5              GTGAAGGCCTATCTTTCTCTCCACCCTCAGGTTTTAGATGAATTTGTGTCTGAAAGCGTT 258
                ************************************************************

clone           AGTGCAGAGACTGTAGAAAAAATGGCTGAAGCGGAAAAACAAGAAGTCAGAAGATGAATCA 415
X5              AGTGCAGAGACTGTAGAAAAAATGGCTGAAGCGGAAAAACAAGAAGTCAGAAGATGAATCA 318
                ************************************************************

clone           GCTCCTAAGGAAGTCAGCAGGTATCAAGATACAAATATGCAAGGAGTTGTGTATGAACTA 475
X5              GCTCCTAAGGAAGTCAGCAGGTATCAAGATACAAATATGCAAGGAGTTGTGTATGAACTA 378
                ************************************************************

clone           AATAGCTATATAGAACAGCGGTTGGATACAGGAGGAGACAACCAGCTACTCCTCTATGAA 535
X5              AATAGCTATATAGAACAGCGGTTGGATACAGGAGGAGACAACCAGCTACTCCTCTATGAA 438
                ************************************************************

clone           CTGAGCAGCATCATCAAAATCGCCACAAAAGCTGACGGATTTGCACTGTATTTCCTTGGA 595
X5              CTGAGCAGCATCATCAAAATCGCCACAAAAGCTGACGGATTTGCACTGTATTTCCTTGGA 498
                ************************************************************

clone           GAGTGCAATAACGAGTGTCAAATTATCTAATCTGCCATATTGCTTGCAACAGATGTTGAC 655
X5              GAGTGCAATAAC------------------------------------------------ 510
                ************

clone           ATACTTCTAAAATAGCCTTTGTGTGTTTATCCCGCCTGGAAT 697
X5              -------------AGCCTTTGTGTGTTTATCCCGCCTGGAAT 539
                             *****************************
```
